# Inferring Species Richness and Turnover by Statistical Multiresolution Texture Analysis of Satellite Imagery

**DOI:** 10.1371/journal.pone.0046616

**Published:** 2012-10-24

**Authors:** Matteo Convertino, Rami S. Mangoubi, Igor Linkov, Nathan C. Lowry, Mukund Desai

**Affiliations:** 1 Risk and Decision Science Team, Environmental Laboratory, Engineering Research and Development Center, United States Army Corps of Engineers, Concord, Massachusetts, United States of America; 2 Department of Agricultural and Biological Engineering - Institute of Food and Agricultural Sciences, University of Florida, Gainesville, Florida, United States of America; 3 Florida Climate Institute, University of Florida-Florida State University, Gainesville, Florida, United States of America; 4 Algorithms and Software, Charles Stark Draper Laboratory, Inc., Cambridge, Massachusetts, United States of America; 5 Department of Engineering and Public Policy, Carnegie Mellon University, Pittsburgh, Pennsylvania, United States of America; University of Western Australia, Australia

## Abstract

**Background:**

The quantification of species-richness and species-turnover is essential to effective monitoring of ecosystems. Wetland ecosystems are particularly in need of such monitoring due to their sensitivity to rainfall, water management and other external factors that affect hydrology, soil, and species patterns. A key challenge for environmental scientists is determining the linkage between natural and human stressors, and the effect of that linkage at the species level in space and time. We propose pixel intensity based Shannon entropy for estimating species-richness, and introduce a method based on statistical wavelet multiresolution texture analysis to quantitatively assess interseasonal and interannual species turnover.

**Methodology/Principal Findings:**

We model satellite images of regions of interest as textures. We define a texture in an image as a spatial domain where the variations in pixel intensity across the image are both stochastic and multiscale. To compare two textures quantitatively, we first obtain a multiresolution wavelet decomposition of each. Either an appropriate probability density function (pdf) model for the coefficients at each subband is selected, and its parameters estimated, or, a non-parametric approach using histograms is adopted. We choose the former, where the wavelet coefficients of the multiresolution decomposition at each subband are modeled as samples from the generalized Gaussian pdf. We then obtain the joint pdf for the coefficients for all subbands, assuming independence across subbands; an approximation that simplifies the computational burden significantly without sacrificing the ability to statistically distinguish textures. We measure the difference between two textures' representative pdf's via the Kullback-Leibler divergence (KL). Species turnover, or 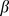 diversity, is estimated using both this KL divergence and the difference in Shannon entropy. Additionally, we predict species richness, or 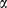 diversity, based on the Shannon entropy of pixel intensity.To test our approach, we specifically use the green band of Landsat images for a water conservation area in the Florida Everglades. We validate our predictions against data of species occurrences for a twenty-eight years long period for both wet and dry seasons. Our method correctly predicts 73% of species richness. For species turnover, the newly proposed KL divergence prediction performance is near 100% accurate. This represents a significant improvement over the more conventional Shannon entropy difference, which provides 85% accuracy. Furthermore, we find that changes in soil and water patterns, as measured by fluctuations of the Shannon entropy for the red and blue bands respectively, are positively correlated with changes in vegetation. The fluctuations are smaller in the wet season when compared to the dry season.

**Conclusions/Significance:**

Texture-based statistical multiresolution image analysis is a promising method for quantifying interseasonal differences and, consequently, the degree to which vegetation, soil, and water patterns vary. The proposed automated method for quantifying species richness and turnover can also provide analysis at higher spatial and temporal resolution than is currently obtainable from expensive monitoring campaigns, thus enabling more prompt, more cost effective inference and decision making support regarding anomalous variations in biodiversity. Additionally, a matrix-based visualization of the statistical multiresolution analysis is presented to facilitate both insight and quick recognition of anomalous data.

## Introduction

### Background

Recent decades witnessed a considerable number of alien species being brought into wetland with significant impacts on local ecosystem structure and biodiversity richness across taxa [1]. In particular, variation in hydrological regimes that occur due to natural and anthropic factors strongly affect water-dependent ecosystems [2], [3], [4]. Wetlands are particularly fragile due to host species' high sensitivity to variations in water regime fluctuations. In fact, wetlands respond to nutrient enrichment of associated waters in typical fashion [5]: some shift in plant community composition occurs first after nutrient levels in soil increase, followed by changes in both aquatic and wetland-dependent animal communities [6]. In oligotrophic wetlands, such as peatlands and ombrotrophic bogs, responses to eutrophication may be more rapid, more dramatic, and longer lasting, naturally implying an intrinsic strong correlation between water, soils, and vegetation [7], [5]. Particularly not well understood is the correlation between species richness and rainfall [8], [2]. Yet, an understanding of linkages between soil properties in wetlands and above-ground landscape patterns is critical to developing quantitative soil-landscape models that will aid in detecting, localizing, and characterizing changes in species composition.

Currently, assessment of biodiversity at local and regional scales often relies on fieldwork-based data collection [9]. Species assessment in relatively large or weakly accessible areas is a longtime challenging task for ecologists. As stressed in [10], a key factors need to be determined before a sampling design is ready for implementation, such as: (i) the number of sampling units; (ii) the spatial placement of the sampling units; (iii) clear definition of the statistically meaningful species of concern; and, (iv) an operational definition of a species community. Moreover, field-based approaches are typically labor intensive and costly, and only a small fraction of a study area may be sampled [10]. The same holds true for hydrological and geological fieldworks that aim to better understand the dynamics of water and soils.

The above discussion attests to a crying need for methods that extract information and accurately analyze remote sensing images nowadays available. High-resolution satellite imagery provides detailed spatial characteristics over large areas of ecosystems and offers a promising potential for accurate vegetation mapping [11], [12], [13], [14], [15]. However, most multispectral image classification techniques more commonly focus on spectral discrimination of ground objects for single-species detection [16], [17], and may overlook pertinent information extractable through analysis of spatial, indeed spatiotemporal, pixel intensity variation residing in high resolution images [18], not to mention the multiscale nature of these variations. Noteworthy, the work in [18] emphasizes the need for methods that rapidly and objectively forecast species diversity thru spatiotemporal data analysis. The work in [18] also demonstrates how previous techniques produced poor assessment of species-richness, or 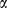 diversity, and particularly pairwise species dissimilarity, or 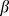 diversity. While the work in [19] focuses attention on 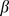 diversity as an important measure of species dissimilarity between communities, the spatial analysis presented do not consider the time varying aspects of species dissimilarity. Besides the pairwise species dissimilarity, species-turnover, or 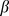 diversity, reflects the change of environmental variations, such as of rainfall and soil, as dictated by either natural events, anthropic events, or both [2]. Species turnover is a major motivation for the work in [20], [21], and a significant number of research efforts analyze 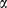 diversity at different spatial scales (e.g. [22], [23], [24], [8], [25], [26]). Yet, apart from [10], time variations in species composition is often overlooked.

### Greater Everglades Ecosystem Restoration

**Figure 1:**
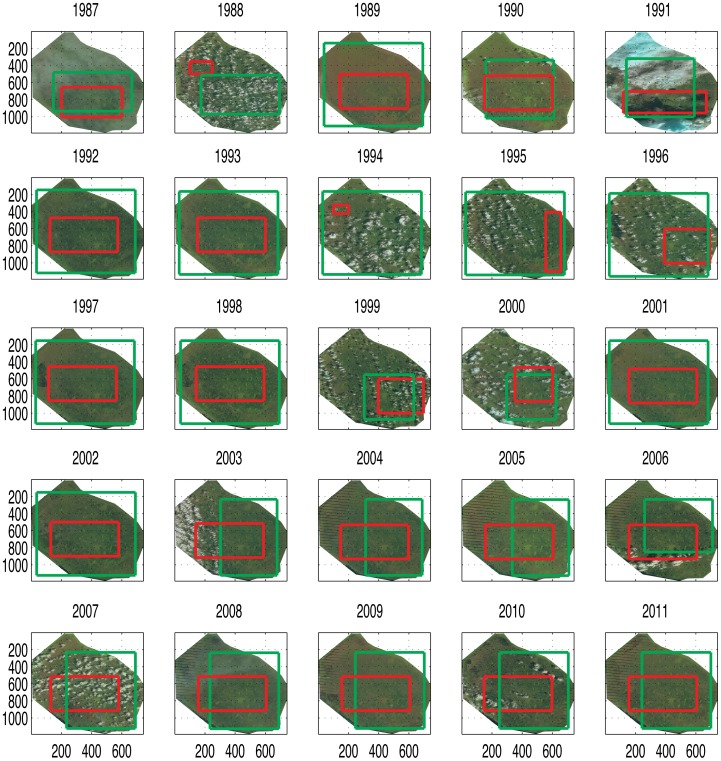
Remote-sensed images for the Arthur R. Marshall Loxahatchee National Wildlife Refuge (WCA 1) during the dry season for the period 1987–2011. The first three years (1984–1986) images are not represented. The representative region in which the texture analysis is performed is delineated in red for each image. The red regions are characterized by a cloud cover lower than 20%. The green regions identify where the data of species are available. Figure S1 reports the images for the wet season.

In this study we consider the Water Conservation Area 1 (WCA 1) in the Greater Everglades Restoration Area, also known as the Arthur R. Marshall Loxahatchee National Wildlife Refuge. This is a constructed tropical wetland (Figure 1). We demonstrate our approach for 28 year wet and dry seasons using Landsat observations. Among wetlands worldwide, the Greater Everglades Ecosystem Restoration area (GEER) has undergone the most considerable changes in ecohydrological patterns. The changes are due to the constructions of a set of levees and canals aimed to control water flow in the area. Thus, GEER is a reference wetland among ecohydrologists and environmental scientists at large [7], [1], [27], [4], [3]. The work in [4] analyzes the effect of rainfall variation induced by climate change for the whole Everglades National Park. This study evidenced the importance of rainfall for the Everglades, an ecosystem defined as strongly rainfall-dominated. Within GEER, WCA 1 is one area that, in comparison to others, underwent the smallest ecohydrological changes; rainfall thus controls the majority of its water. Thus, WCA 1 is a unique area to test the correlation between climatological and ecohydrological patterns. The choice of GEER to demonstrate our proposed methods is opportune, as there is currently a serious ongoing debate regarding the restoration of Everglades according to the original predrainage patterns; the main concern regards effects that such restoration may bring to the ecosystem [1], [27]. Prior to undertaking such restoration it is very important to develop analytical methods and tools capable of characterizing and localizing multiscale spatiotemporal changes, such as in vegetation patterns, as a function of changes that derive from previous interventions and/or natural changes of climate. Additionally, the analysis of vegetation, plant species-richness in space and time in particular, is also crucial to the development of models that are capable of predicting scenarios of different management alternatives. Management alternatives for WCA 1 are different configurations of the levee system that regulates the ecohydrology in the area. Generally, ecological models are calibrated and validated on observed data, and accurate parameter estimates are therefore critical to enabling these models. Regarding WCA 1, we are only aware of the effort of [28] for evaluating the restoration of the Everglades using satellite imagery. However, [28] did not consider any species richness indicator.

### Approach

In a new approach to this problem, we aim to extract species richness in space and time from multispectral satellite imagery, using statistical multiresolution wavelet texture analysis. We adapt the approach in [29], used for retrieval, to texture classification. We demonstrate our analysis of WCA 1's ecohydrological patterns by considering interseasonal and interannual species dissimilarity - which is better known as “species-turnover” [21], [20] - and species richness for twenty-eight years of Landsat observations. The approach consists of applying wavelet decomposition, and statistically modeling then comparing these coefficients either thru a non-parametric histogram, or thru an appropriate probability density function. We elect a model that embraces a wide spectrum of central and tail behavior that can be represented by varying just two parameters at each subband. Specifically, the coefficients from each scale, even each subband of the decomposition are considered samples from a generalized Gaussian (GG) probability density function (pdf). The coefficients provide estimate of the characterizing GG pdf parameters. We next assume independence across scales and across subbands within scales and obtain a joint pdf for each textured image region. The independence assumption is only an approximation, but experience has shown that it provides accurate results when the objective is to quantitatively examine differences across regions and time points. There are many ways to quantitatively assess differences between pdf's; we choose the relative entropy, known as the Kullback-Leibler divergence (KL). Other methods, such as likelihood ratios, and various metrics, could also be examined. We use the KL divergence and the Shannon entropy to assess species dissimilarity in time (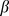 diversity), and species richness (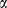 diversity) respectively. Entropy is widely reported as a proxy of species richness [30], while spectral heterogeneity measured by reflectivity is a measure to quantify the entropy [31], [32].

We validate our predictions on independent data from the Global Biodiversity Information Facility [33] that are composed by field-data. We do not compare directly our method with other multispectral analysis methods (e.g. see [18] for a review) as we prefer to provide a novel theoretical and methodological framework - tested against long duration data records - for analyzing multispectral images.

### Previous Research

To the best of our knowledge this is the first time that a statistical model based multiresolution texture analysis is applied to satellite imagery for quantifying species-turnover, a multiscale spatiotemporal phenomenon. Previously, the work in [19], [34] and recently [35] used the KL divergence for estimating 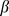 diversity in space as a theoretical construct without any model. In [36], texture analysis predicts avian biodiversity richness based on satellite imagery. Species turnover, however, was not considered. Other previous texture-based calculation used the intensity of imagery for estimating biodiversity variables. Texture-based analysis have investigated species habitat relationships [37], successional attributes of forest species [38], classification of species [39], species richness as a function of spatial and spectral resolution [15], intercorrelations of vegetation biodiversity and soil dynamics at both local and regional scales [40], and different band combinations of images [41].

None of the above authors, however, specifically account for multiple scale variations in both space and time. Statistical multiresolution texture analysis proved successful in other applications; it is previously applied to classify stem cell colonies [42], [43], [44], thus enabling non-invasive analysis of these cells. Non-invasive analysis of cells is a disruptive technology that carries the potential of supplementing, even replacing invasive and at times destructive chemical biomarkers. As such, in addition to lowering cost, it provides two advantages: (1) it provides a quantitative statistical assessment of these cells; and (2) enables the preservation of these cells for their intended use, such as drug testing and tissue formation. For the ecological study of concern here, the analysis will have similar advantages: it will reduce the need for costly field fieldwork-based data collection [9]. It is also non-invasive, while fieldwork data may also perturb the ecosystem and the reliability of the collected data may be low because of the generated disturbance or because of the limitedness of the sampled data [45], [1].

### Summary of Contributions

**Figure 2:**
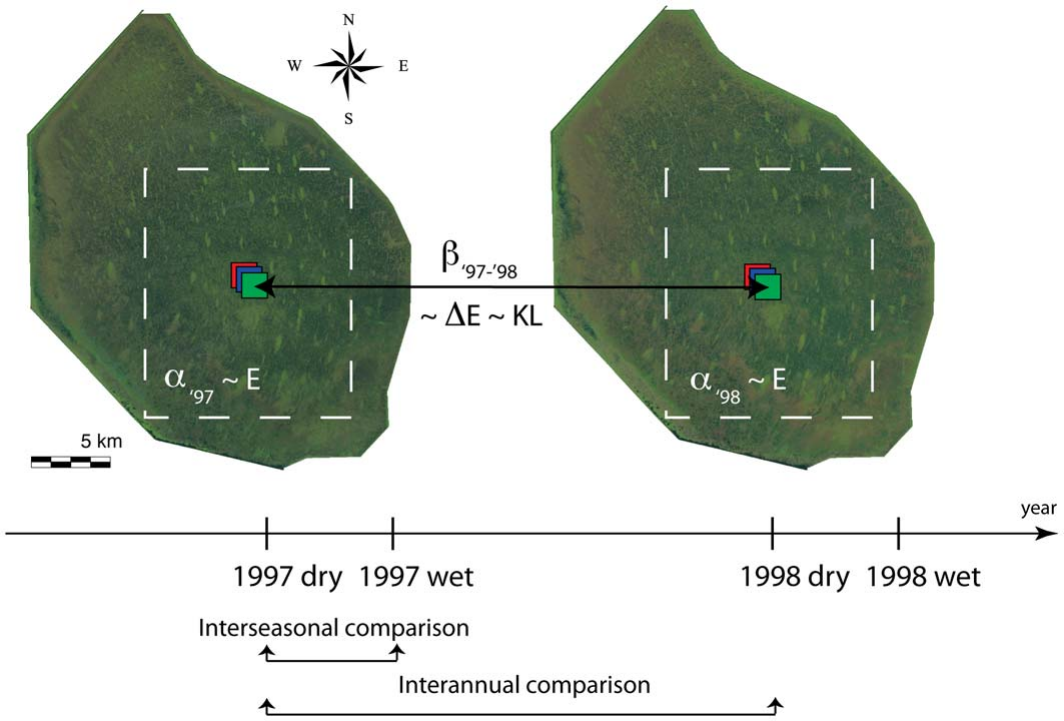
Graphical explanation of the analysis performed for the WCA 1. Landsat images (from 1984 to 2011) are acquired for the dry and the wet season. The example is reported for the years 1997 and 1998. The changes in vegetation composition are analyzed using 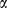 and 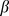 diversity among seasons and among years. For the interseasonal analysis the time-scale is on average six months between seasons of the same year, while for the interannual analysis the time-scale is about a year between the same season of different years. 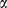 and 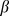 diversity from data are compared to the estimates of the Shannon entropy (Equation 5) and of the KL divergence (Equation 4) for the green band of the images respectively.

The overall objective of this study is to investigate the spatiotemporal relationships between soil, water, and vegetation patterns derived from remote sensing data in a tropical wetland. We adapt and apply a multiresolution statistical texture analysis to remote sensing imagery. The methodology developed aims to account for both the statistical and multiscale spatiotemporal nature of the above mentioned relationships, thus enabling more parsimonious inference at higher spatial and temporal resolution. We also investigate the accuracy of the KL divergence as a measure of 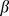 diversity in time versus conventional approaches such as the difference of the Shannon entropy. We detect: (i) the hydrological footprints in term of correlation between water, vegetation, and soil changes; (ii) the interseasonal variation of species-richness in which dry and wet seasons are compared for each year (Figure 2); and, (ii) the interannual variation of species-richness for the same season between consecutive and non-consecutive years (Figure 2). We verify that the use of the KL divergence estimated on texture performs better than conventional methods. This analysis is performed successfully on low-medium resolution data (Landsat images). Moreover, we illustrate the results with a novel color based visualization that provides insight into the ecological variations, and enables a quick recognition of anomalous data.

## Materials and Methods

### Satellite Imagery

The satellite images of the Water Conservation Area 1, described in Figure 1 and Text S1, are derived from the Landsat database of [46] and [47]. Figure 1 and Figure S1 show WCA 1 for the 1987–2011 period in the dry and wet season respectively. The resolution of these images is 30 m. The images from 1984 to 1998 are from the L4–5 TM dataset, from 1999 to 2003 from the L7 ETM+ with SLC-on (1999–2003), and from 2003 to present from the L7 ETM+ with SLC-off. The Scan Line Corrector (SLC) stopped working on May 31, 2003 and that caused striping of remote sensed images [48]. Thus, we do not consider in our analysis the striped parts of these images. Each Landsat image is cropped along the boundaries of the Water Conservation Area 1 provided by the South Florida Water Management District. The representative area of analysis for each image is selected in oder to guarantee a cloud cover lower than 20% on average for each selected area and a minimum area requirement for the texture method (Section). At the same time, we strived to maximize the extension of the selected area and its representativeness of the whole landscape. For each year two images are collected, one for the dry season, December to April, and one for the wet season, May to November. Images were selected in January and August, the most representative months for the dry and wet season respectively. In Figures 1 and S1, the red squares show the regions selected for texture analysis. We analyze the satellite images for each visible band defined as:

We analyze the satellite images for each visible band defined as:

- Band 1: (Blue; electromagnetic wavelength: 0.45–0.52 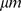), which is useful for mapping *water*, differentiating between soil and plants, and identifying manmade objects such as roads and buildings;
- Band 2: (Green; electromagnetic wavelength: 0.52–0.60 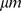), which spans the region between the blue and red chlorophyll absorption bands, and shows the green reflectance of *healthy vegetation* (thus vegetation that changes across seasons and years). It is useful for differentiating between types of plants, determining the health of plants, and identifying manmade objects;
- Band 3: (Red; electromagnetic wavelength: 0.63–0.69 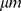), which is one of the most important bands for discriminating among different kinds of vegetation. It is also useful for mapping soil type boundaries and geological formation boundaries. It is considered as a proxy of *soil* types.

### Species Occurrences

The species richness for WCA 1 is compiled by assembling together data from fieldworks of [45], the Global Biodiversity Information Facility database [33], and the Comprehensive Everglades Restoration Project database [49]. In order to compare the predicted species richness from the analysis of the green band of Landsat images, we build a species richness matrix at 30 m resolution from the aformentioned sources. For more information about WCA 1 we refer the reader to Text S1.

The “base” local species-richness (or 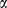 diversity) of plant communities is built from the Global Biodiversity Information Facility [33] data that provide occurrences of species in space and time at 1 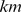 resolution. The green squares in Figure 1 and S1 indicate the regions where the GBIF data are available. We consider data from 1984 to 2011 such as for the Landsat images. For the GBIF data we downscale the information of species richness from 1 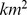 to 30 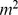 using a simple coarse-graining algorithm [50]. A grid of smaller boxes is created and the number of species are counted at the resolution of the smaller grid. The estimation of species richness is also refined with point data of plant species occurrences from CERPzone, that is the database of the Comprehensive Everglades Restoration Project [49]. CERPzone data are available from 2001. In the species richness matrix we also include the data of [45], where a total of 30 plant species are found along the Loxahatchee National Wildlife Refuge transect which goes from the westernmost to the easternmost boundary of WCA 1 at its maximum width. According to [45] the species-richness at a given site never exceeded 8 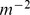. Of these 30 species, only 11 were found in both 1989 and 1999 (Table 2 in [45]).

The occurrences of these three datasets are compared together after creating a grid over WCA 1 in which the unitary pixel is characterized by an area of 30 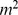. The point occurrences of CERP and [45] are downscaled to 30 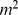 following the approach of [51] that is a shot-noise Cox process method. All the unique occurrences of species from all datasets are assigned to each pixel according to a distance criterion. Each occurrence characterized by geographic coordinates is assigned to the nearest neighbor pixel. The local species-richness or 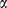 diversity is calculated by the sum of unique species in each pixel, and the average local species-richness of WCA 1 is the average over all the species-richness values of each pixel. Species turnover or 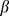 diversity is calculated on the data of [33] as complementary to the Jaccard Similarity Index (JSI) evaluated in time at resolution of 30 m. JSI is given by the ratio of the number of common species in two pixels considered and the number of all species in both pixels. Specifically, 
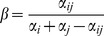
, where 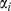 and 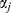 are the numbers of species present in pixel 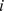 and 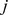 at different seasons or years). We also note that 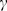 diversity is the number of distinct species in the whole domain of the green squares (Figure 1).

### Specific Study Hypothesis

Our study is based on the following hypothesis formulated on some previous studies and data.

1. Rainfall is the main driver of vegetation patterns in WCA 1 and in tropical wetlands [1]. This is evidenced by hydrologic studies [4] and by analysis of satellite imagery pre- and post-construction of the levee-canal drainage systems [1] We do not anticipate any relationship between changes in soil, water, and vegetation richness in WCA 1 because non-linearities were evidenced among these variables [5]. However, because of the high seasonality of climate [8], [1] we expect variation of species richness within each year for the wet and dry season such as was reported for single species [52].
2. Landsat RGB imagery, despite its low resolution,reveals information about the spatiotemporal structure of ecosystem components [53], [54]. As stated by [54] high resolution imagery is not always available for free for all the regions in the world. Moreover, imagery of higher quality does not always guarantee better estimation of biodiversity variables. This is particularly true in tropical ecosystems where the resolution of high quality imagery can be too small compared to the extent of each single species [54]. We believe that the improvement of methodologies for assessing biodiversity variables and other environmental variables of ecosystems such as in this paper, can overcome the low quality of imagery such as the Landsat imagery.
3. The Shannon entropy of the green band can be considered as the spectral heterogeneity (reflectance) of plant species in water-dominated ecosystems [55], [56], [57], [58], [59], [60], [61], [62], [63], [64], [65], [66]. The blue and red bands can be considered as the spectral signatures of water and soil heterogeneities in the ecosystem [55]. Different species have different reflectance levels for different bands of the Landsat images. The higher the range of reflectivity, the higher the entropy because there are potentially more plant species with different degree of reflectivity. In ecology there is an large discussion that different reflectance levels do not always characterize different species, but “functional species” [55], [58], [59], [67], [53], [68]. The issue about species diversity versus functional diversity is a longstanding issue in ecology. Functional diversity, which is different but related to taxonomic diversity (i.e., species richness), is crucial to ecosystems and the services they provide to humans [59]. In this paper we talk about “species” rather than “functional species”; nonetheless, we make clear that similar reflectance levels may characterize functional species or vegetation types (within the same spectral group) rather than the same species. No distinction is made here between invasive and endemic species; however, the variation of species richness can also be attributed to the invasion of non-native species and inform species management. Other sources of variation in surface reflectance, such as directional effects and shadowing, are also sources of texture variations in Landsat scenes. Nonetheless, we believe our algorithm is capable of detecting the average texture generated by species differences.

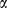
 and 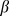 diversity are related to the Shannon entropy and to the KL divergence of the green band respectively. Previous studies investigated these relationships based on data and theoretical constructs [19], [35]. We also hypothesize that 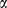 and 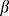 diversity in time are independent measures of biodiversity richness as suggested by previous studies [69], [70], [71], [72], [73], [20]. We conjecture that the average 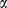 diversity is a lower but close estimate of 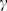 diversity. This is because the distribution of species is quite homogeneous within WCA 1 as verified by data [33] and as reported in [74]. Thus, because the number of species is on average the same in any representative region within the whole ecosystem and for the period considered, scale-invariance of 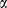 diversity is hypothesized.
5. The difference between Shannon entropies provides a worse estimate of 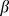 diversity in time than the KL divergence. We expected that this occurs because the KL divergence, that is not calculated on the intensity of the Shannon entropy, potentially accounts for the pairwise interactions between vegetation communities in time (i.e. the mutual information in information theory). On the contrary, the difference between Shannon entropies does not capture the community pairwise interactions [19].

### Texture Image Analysis

We model the region of interest as textures. Texture models abound in the literature and textbooks (see e.g. [75]). We favor a model that captures what in our view are two essential properties of texture: variations of intensity across pixels that: (i) occur at multiple scale, and (ii) are stochastic in nature. Such a model suggests that a wavelet analysis can decompose the spatial intensity variation into multiple scales, and subbands within scales, after which the coefficients of the decomposition at each scale can be analyzed statistically. The across scale statistical analysis characterizes the texture. This is the approach followed in [29] for texture retrieval. As wavelet analysis decomposes a signal locally according to orientation and scale, it is especially apt for modeling texture, characterized by intensity randomness at multiple scales. Note that perceived image texture is closely related to the degree of random fluctuation in image gray-scale intensity at multiple frequencies. An 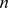-level wavelet decomposition produces 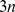 detail subbands, three per level, whose coefficients convey information about the fluctuation at a particular scale and orientation (one oriented horizontally, one vertically, and one diagonally). We compute textural features from these subbands by modeling the empirical pdf of the coefficients in each subband. Moving from one level to the next the scale increases. The above method was also adapted to texture comparison and classification, and used to non-invasively and quantitatively classify stem cell colonies [42], [43], without using chemical markers, thus preserving the colonies for use. The method is also used to classify nuclei in [44], [76], [77]. We will adopt this method here to compare Landsat images of regions across seasons and years, and demonstrate in the next section that it successfully predicts species turnover. A complete description of this approach is beyond the scope of this paper, byt the approach is thoroughly developed in [29] and [42]. We briefly outline the steps below. For representing a texture by a probability model:

#### 1. Texture window selection

For the texture of interest within the image, select the largest possible representative window where the patch (or region) of the image is analyzed; a minimum of 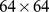 pixels is required. The patches have different size from year to year. The size of the patches varies because we aim to select the largest cloud-free regions possible. Thus, size is increased until the cloud coverage in the area is lower than 20%. We used a moving window in space to select each patch. The size of the patches varies from 10% to 96% of the total area of WCA 1 that is 588 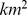 (Figure 1). Thus the area of the patches varies from 58 to 564 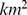 approximately.

#### 2. Wavelet decomposition

Apply a multiscale wavelet decomposition to the selected window. Collect the coefficients at each detail scale of the decomposition. The specific choice of wavelet and the number of scales is a design choice, and more than one choice will yield appropriate representations. Wavelet analysis is a generalization of Fourier analysis that quantifies the degree at which pixel intensity varies at multiple scales or electronic magnifications. As wavelet analysis decomposes a signal locally according to orientation and scale, it is especially apt for modeling texture, characterized by intensity randomness at multiple scales. Note that perceived image texture is closely related to the degree of random fluctuation in image gray-scale intensity at multiple frequencies. An 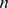-level wavelet decomposition produces 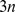 detail subbands, three per scale, whose coefficients convey information about the fluctuation at a particular scale and orientation (one oriented horizontally, one vertically, and one diagonally). We compute textural features from these subbands by modeling the empirical pdf of the coefficients in each subband. Moving from one level to the next the scale increases.

#### 3. Probabilistic modeling at each scale

For each decomposition scale, either represent the coefficients by a non-parametric histogram, or instead select a probability density function (pdf) as a model and use the coefficients to estimate the pdf's parameters. In [76], the first approach, non-parametric histograms, is used, while in [29], [43], [44], [77], the Generalized Gaussian (GG) probability density function was selected as an appropriate model for the wavelet coefficients at each scale. The GG pdf for detail coefficients at scale s is given by:
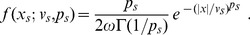
(1)Here, 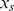 is the random variable or detail coefficient at scale 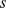, and 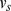 and 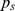 are the distribution width factor and shape parameter, respectively, for subband 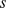. Additionally, 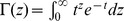 indicates the Gamma function. We assume the location parameter (i.e. process mean) to be zero as the detail coefficients are the outputs of a high-pass filters. The GG density may be used to model a wide variety of symmetric, unimodal density functions; special cases include the (standard) Gaussian ((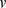, 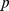) =  (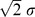, 2)), standard Laplacian ((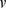, 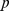) = (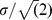, 1)), and uniform (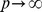) densities. 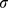 is the standard deviation, which for a GG Distribution process (GGD) is 
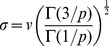
. A variety of techniques exist for estimating parameters 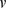 and 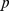, including moment-matching [78] and maximum-likelihood [29], [79], [76].

#### 4. Texture joint pdf model

Construct the joint pdf for the texture by combining the pdf's for each scale's subbands, assuming independence across scales. We note that such an assumption is not true in reality, but it eases the computational burden while providing good results for classification. For 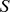 scales, with 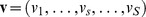, and 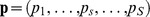.
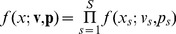
(2)


One of the key advantages of the GG distribution model is that a closed-form solution exists for the KL between two GGD processes simplifying computation: 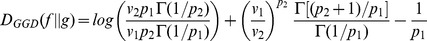. Textures are compared in a pairwise fashion by obtaining the difference between their respective pdf's. Specifically, to compare two textures with pdf's 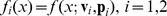, respectively, we calculate a measure of the pdf's divergence or difference. There are many ways to express the divergence between pdf's, including likelihood functions, various metrics such as the L1 divergence (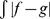), the Bhattacharyya distance, and the KL divergence. We elect the latter as it is inspired by the relative entropy between pdf's. The information divergence is especially convenient as it admits a tractable closed-form solution for GG distributions. Specifically, we have for the Kulback-Leibler divergence,
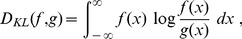
(3)that theoretically is 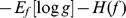 where 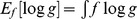 is the expectation under 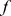 of 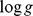, and 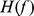 is the true entropy in 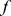, i.e. 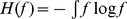. The divergence in Equation 3 is not symmetric, meaning that 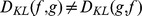. To obtain a symmetric version, we define
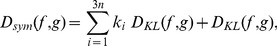
(4)summing across all 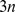 subbands, where 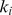 are simply weights assigned to particular subbands 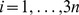. In general, we set all 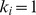, but the weights might be used to emphasize or penalize certain bands according to prior knowledge. In the results and discussion section, we demonstrate that the above symmetrized KL divergence is an effective predictor of species turnover.

### Ecological Equivalence

The ecological equivalence of texture analysis is based on the assumption that the entropy and the KL divergence represent the potential species-richness and the dissimilarity in time of species-richness within communities (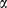 and 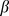 diversity respectively). In the following we propose a theoretical description of entropy and KL divergence in ecology making analogies to the definition of these quantities in image analysis (Section). Because of our hypothesis on the reflectance of species, the entropy in shades of green means diversity in types of plants, which determines 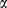 diversity. KL divergence between two regions at different times means different shades of green for the two regions, which determines 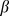 diversity in time. 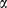 and 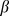 diversities are among the most employed theoretical concepts in ecology and biodiversity conservation. For most ecologists, 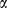 diversity traditionally reflects the within-habitat diversity [80], whereas 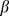 diversity is the component of “total diversity” that is produced by differences in species composition among the sampling units [81]. The need to partition diversity within and among habitats has both theoretical and applied interests. Species-richness is a diversity index of order zero, Shannon entropy is a diversity index of order one, and all divergence measures are diversity indices of order two [30], [18]. Because the Shannon entropy is a first order measure of species diversity [30], the variation of Shannon entropy in time is as well a first order measure of species diversity between the same or different regions.

The Hill order [82] is the order of any information measure that is derived from the generalized expression 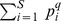 or limits of such functions as 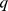 approaches unity, where 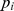 is a probability distribution function and 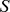 is the number of equally common species that compose a community. The diversity metrics from 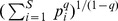 are often called “Hill numbers”, but they are more general than [82]'s derivation suggests [30]. The exponent and superscript 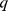 may be called the “order” of the diversity; the true diversity depends only on the value of 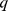 and the species frequencies, and not on the functional form of the index. For 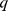 that approaches unity the information functions are the species richness, Shannon entropy, all Simpson measures, all Renyi entropies, all HCDT or “Tsallis” entropies, and many others. All values of 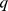 less than unity give diversities that disproportionately favor rare species, while all values of 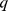 greater than unity disproportionately favor the most common species [83], [30].

Generally the higher the metric's order the better the estimation of the ecological metric. With the knowledge of entropy the species-turnover could be potentially calculated as difference of entropies between two different time steps. However, we consider whether the KL divergence is a better measure with respect to the difference of entropies. In fact, the KL divergence captures the pairwise variation of species-richness of local communities and not only their independent variation of species-richness [19]. For example [84] showed how the pairwise interaction is not negligible in grasslands and this has profound implications for the ecosystem functioning. Recently the KL divergence was related to the Shannon entropy and the species-richness in the whole ecosystem analyzed [35]. We consider that the species-richness and dissimilarity estimations are representative of the whole region extending the analysis from the subregions in which the texture analysis is performed. The entropy calculated by our texture analysis method can be assumed to be an estimate of the maximum likelihood estimation of the Shannon's index [19] defined as:
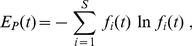
(5)where 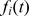 is the observed sample fraction a local community 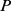 (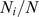, where 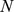 can be considered as the number of individuals of species 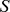, and 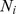 is the number of individuals of the 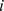-th species) at time 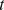. The entropy here introduced has the same meaning of the entropy calculated using the intensity of the green band images. The same entropy can be calculated over an other local community 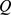 for an other function 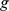. The entropy as defined above is one of the most commonly used index of 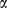 diversity [19], [71], that is the number of species in a subarea of the region considered, in theoretical ecology and ecological modeling. Certainly there are other metrics to estimate 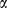 diversity, but the entropy is one of the most profound and useful of all diversity indices. However its value gives the uncertainty rather than the diversity. Thus, the entropy needs to be rescaled to diversity given some data or calculated as in [30]. The entropy is easily derived from the “order generating function” of [81]. The predicted 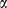 diversity is tested vs. the estimation of 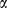 diversity from the data described in Section at resolution of 30 m.

The KL divergence calculated by our texture analysis method is an estimate of the divergence:
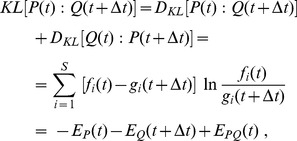
(6)where 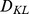 is the discriminant information introduced by [85] (Equation 3). Thus, KL is the symmetric version of the divergence 4. Equation 6 is estimated using Equation 4. Let`s assume that local communities (or regions) 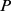 and 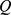 are composed by the same species (labelled from 1 to 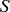) with probability distributions 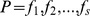 and 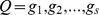 respectively, with 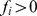, 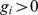 and 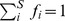, 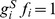. 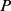 and 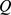 are evaluated at different time step, 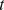 and 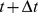 where in our analysis 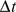 is approximatively six-month and a year for the interseasonal and interannual comparison of the species dissimilarity, respectively. 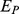 and 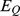 are the Shannon entropies of communities 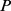 and 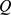 calculated as in Equation 5, and 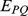 is the “reciprocal information”. The reciprocal information can be assessed only after the knowledge of KL (Equation 4) and of 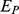 and 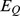. The reciprocal information represents the pairwise interactions among vegetation species in different communities in time (or in space) which is not captured by completely by the differences of entropies of the two local communities. The KL divergence gives a measure of how much the distribution of species within the communities differs from one another. This has been traditionally called 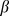 diversity in ecology. The 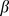 diversity is the most useful metric from which it is possible to assess the number of species and their abundance. We do not use the KL divergence for measuring the dissimilarity of species in space within the same region or between different regions although this is a possible operation that can be performed. Thus, Equation 4 is a proxy of Equation 6 of the landscape analyzed.

## Results

**Figure 3:**
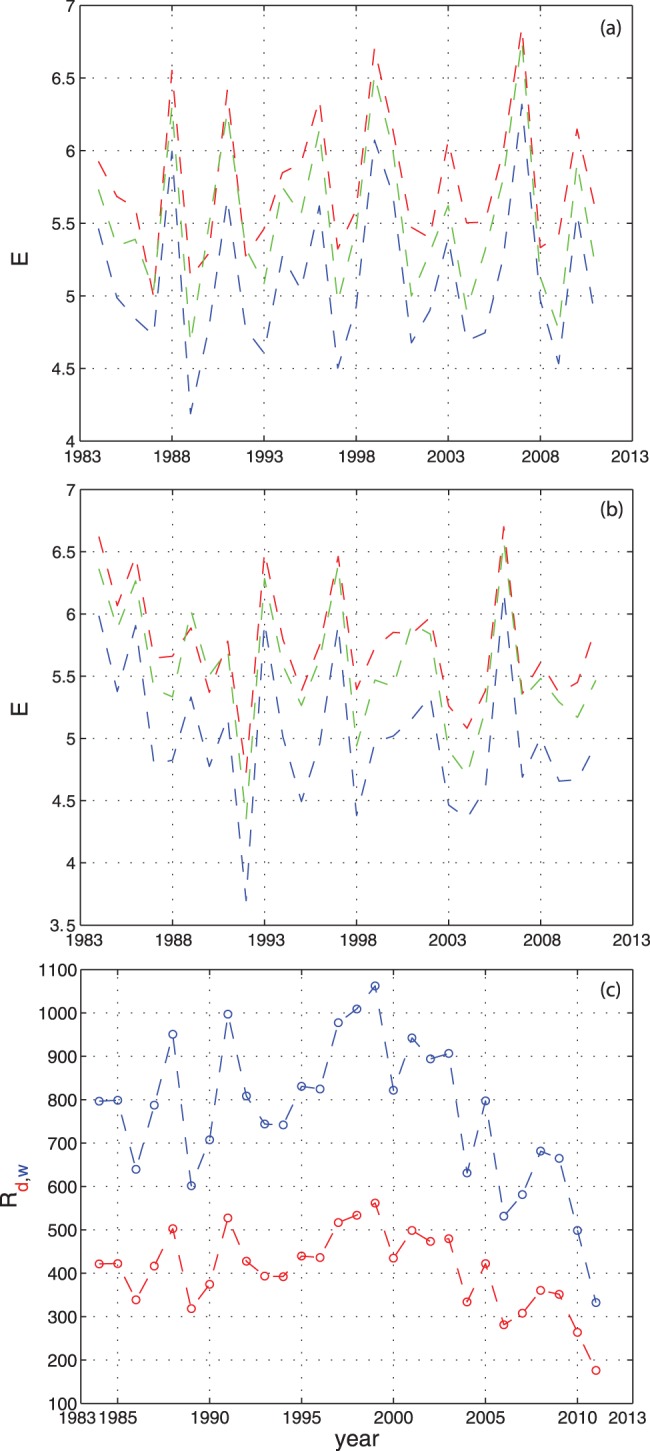
Interannual entropy of WCA 1 Landsat images and rainfall in the period 1984–2011 considered. (a, b) Shannon entropy of the representative regions of WCA 1 in the dry and in the wet season respectively (Figure 1, and Figure S1) for the red, green, and blue bands. The Shannon entropy for the green bad is proportional to the 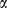 diversity of plant species. (c) Average annual rainfall (in mm) in the dry (red line) and wet season (blue line).

The entropy of each band for the dry and wet seasons is shown in Figure 3. The Shannon entropy is calculated on the green band of the Landsat images using Equation 5. In this paper we use the Shannon entropy of the RGB bands as spectral signature of temporal heterogeneity of the landscape for soil, vegetation and water. Thus, we deviate from [11] which uses the spectral heterogeneity as the mean of the pairwise Euclidean distances in the wavebands dimensional space. There is no significant decadal change in plant community patterns as confirmed by [45]. In fact, the entropy of the green band that is a proxy of 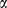 diversity is overall constant both for the dry and wet season in the twenty-eight years analyzed. This may be both related to the confinement of species within WCA 1 (the set of surrounding levees block immigration of species) and to the absence of dominating species that do not spread over large regions. Thus, the set of species living on WCA 1 is believed to be the same on average. The comparison of Figure 3 (a) and (b) shows that the fluctuations of the Shannon entropy for each band in the wet season (Figure 3, b) are moderately smaller than in the dry season (Figure 3, a). Moreover, the fluctuations of the Shannon entropy of the red, green, and blue bands are highly correlated within the same season. We note also that the Shannon entropy of the RGB bands for the dry and wet seasons are inversely correlated. This is expected considering the opposite ecohydrological dynamics in South Florida in the dry and in the wet season.

The dry season is characterized by a lower density of vegetation than the wet season which theoretically increases the probability to detect different species. However, the reflectance of the green band is higher in the wet season but it tends to be more homogeneous than in the dry season. The Shannon entropy can be considered as the probability to detect species in a given area. Thus, the higher the Shannon entropy, the higher the probability to find all the species within the area considered. Figure 3 (a, b) shows that this probability increases during the dry season due to an higher Shannon entropy than the wet season. Thus, reflectance and heterogeneity may plays a contrasting role in assessing local species richness. We observed a considerable turnover of species-richness that is associated to the fluctuation of the average annual rainfall (Figure 3, c). The entropy of the red band is higher in the dry period than in the wet period as well as the entropy of the blue band in the same season. Despite the decrease of the average annual rainfall in the last decade (Figure S2 (b) shows this trend from about the year 2003) the average species-richness is observed to be constant (Figure S2, a). Figure S2, S3, S4 (Text S1) report the relationships between the average Shannon entropy and the average rainfall at the year scale. Figure S2 reports the interannual entropy and the average annual rainfall derived from [86]; Figure S3 shows the cross-correlation between these two quantities; and, Figure S4 shows the functional relationships between the Shannon entropy and the average rainfall at the season-scale. The correlation between the interannual entropy and the average annual rainfall is almost equal to one for a lag equal to zero. Thus, the highest/lowest species-richness is observed for the highest/lowest rainfall respectively. This result shows that the driver of vegetation richness is the average annual rainfall. The nutrient concentration depends on the water depth that depends itself on the rainfall. Fires, are local phenomena that shape vegetation mostly locally and their frequency decrease for lower rainfall. We believe that the variation of Shannon entropy is a first-oder measure of dissimilarity of a quantity evaluated in different time periods. Thus, this explain why the average entropy for all the bands is higher in the dry season in which the biggest landscape heterogeneities are observed. Figure S5 confirms the strong correlation between the Shannon entropy of the red, green, and blue band. We consider the Shannon entropy of the green band (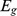) as the dependent variable as a function of water and soil composition that are represented by the Shannon entropy of the blue and red bands respectively. The correlation is very high as evidenced already by the temporal sequences of Figure 3 and Figure S2.

**Figure 4:**
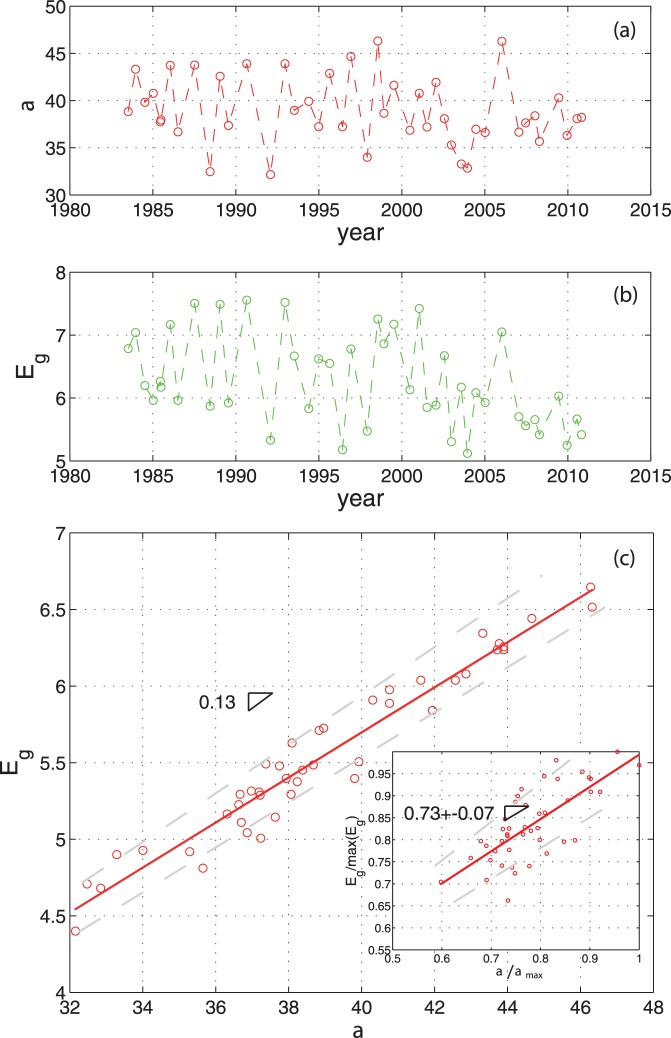
Estimated and measured local species-richness. The local species-richness (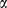 diversity) and the estimated local species-richness (i.e. the Shannon entropy of the green band, 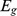 using Equation 5) are reported in plot (a) and (b) from 1984 to 2011 respectively. In plot (c) the functional relationship between the Shannon entropy of the green band and the local species-richness for WCA 1 is reported. The inset, which reports the normalized entropy, shows the ability of the Shannon entropy to capture at least 70% of the measured local species-richness. This percentage is 80% and 77% in the dry and wet season respectively. The dashed grey curves are the 95% confidence interval of the linear regression exponent. Variabilities of measured exponents are found by bootstrapping over points and deriving slopes by the linear and the Jackknife models [92].

The estimated 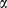 diversity on the data (Global Biodiversity Information Facility [33], [45], and the Comprehensive Everglades Restoration Project database [49]) at resolution 30 m after downscaling of the original data is reported in Figure 4. In 1989, 41 species of vegetation were sampled [45]. According to the Global Biodiversity Information Facility [33] the species were 38. The green band Shannon entropy for that year is equal to six approximatively. Thus, if the entropy is taken proportional to 41 species, we observe that the average number of species fluctuated between 29 (in 1992) and 46 species (in 2006). The potential average 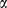 diversity is 38 species. In 1999, 30 species of vegetation were sampled [45]. According to the Global Biodiversity Information Facility [33] the species were 33. In Figure 4 (b) the green band Shannon entropy corresponding to the year 1999 is 5.7 that corresponds to 36 species when rescaled to real diversity [30]. Thus, our estimation of 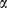 diversity by image analysis is capturing the second observation with an error of three species. Comparing Figure 4 (a) and (b) we observe an high cross-correlation between the measured and estimated 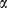 diversity. The inset in Figure 4 (c) shows in fact that the entropy of the green band estimates at least 70% of the measured 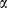 diversity. This percentage is 80% and 77% in the dry and wet season respectively that confirms the assumption of easier detectability of species during the dry season. Although we recognize that the Global Biodiversity Information Facility data [33] is affected by some uncertainty in the estimation of species occurrence [87], we believe that the uncertainty for this area is overall lower due to the extensive sampling campaigns occurred in time (we recorded an average of 20,000 occurrences for each year considering together the data from [33] and [49]). Hence, we believe to have captured the local plant species richness and plant species turnover, that is demonstrated by the excellent similarity between predictions and data. We also believe that the texture estimation algorithm on the red squares in Figure 1 and S1 is a very efficient tool to estimate the species richness of the green squares which are representative of the richness of the whole WCA 1. In our study area because the scale of the patch (or region) considered is very large, and the variation of 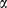 diversity is very low, the maximum 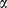 diversity tends to the average 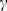 diversity. Because the species are evenly distributed 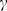 diversity has not a strong dependency to the scale of analysis. Because the reflectance is capable to distinguish different species, the larger the scale the more the estimated 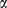 diversity resembles 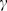 diversity. Gamma diversity is in general greater or equal to the maximum value of 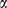 diversity. For ecosystems with very heterogeneous distribution of species this is no more true and local 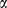 diversity, average 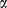 diversity, and 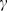 diversity are very different [88].

The average change in species-richness in time is expressed by the variation of the image entropy. The calculated difference between Shannon entropies at different time steps was reported as a worse estimator than the KL divergence of the species-turnover [19]. Nonetheless, It was shown that Shannon's diversity (that is the difference between entropies) is the KL divergence between the actual plot and the “average” plot within the region [35]. A plot is defined as the region where the analysis of biodiversity is performed (e.g. the red squares in Figures 1 and S1). If the average plot (e.g. the green squares in Figures 1 and S1) is sufficiently big to contain the plot considered, the pairwise variation of species-richness due to plot interaction is included in the Shannon diversity. However this is not always the case because disturbances (such as clouds and satellite recognition imperfections) limits the extent of the representative average plot of the region. Moreover it is not easy to define the representative plot due to the intrinsic heterogeneities of the region.

**Figure 5:**
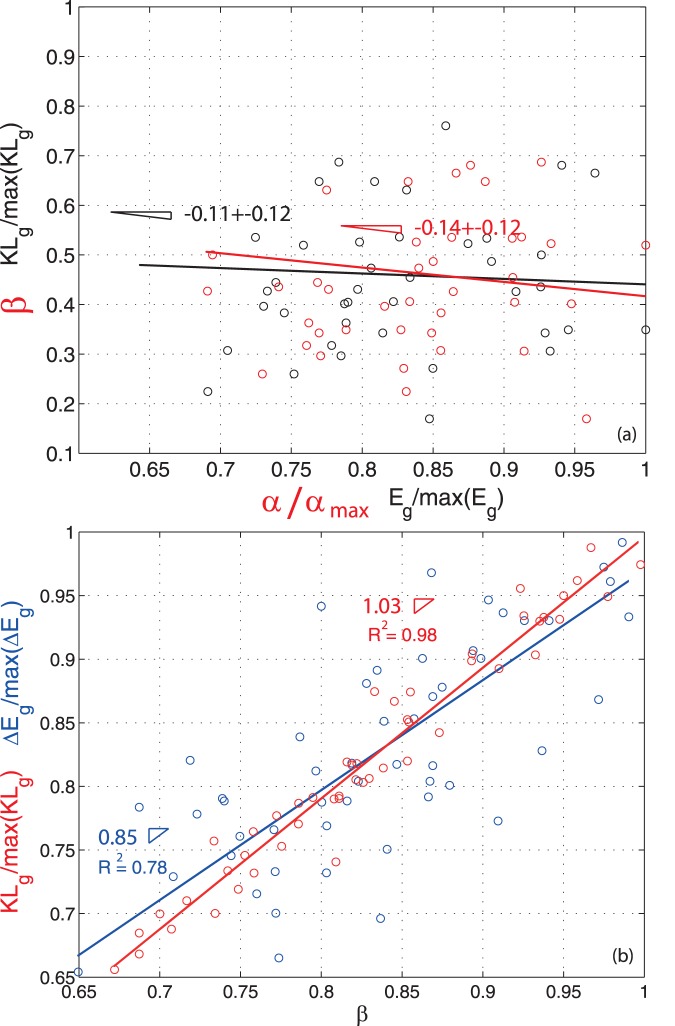
Relationship between 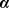 and 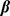 diversity, and estimation of 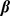 diversity using KL divergence and difference of entropies. (a) Relationship between the interseasonal KL divergence and the green band Shannon entropy for the period 1984–2011, and same relationship estimated on data. The plot shows the independency of 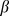 diversity as a function of 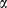 diversity. Plot (b) shows how the KL divergence better predicts 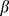 diversity than the difference of the Shannon entropy between seasons. The dashed grey curves are the 95% confidence interval of the linear regression exponent. In (a) the dashed grey curves are the 95% confidence interval of the linear regression exponent. In (b) the error in the estimation of the regression coefficient is 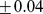. The 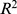 is the coefficient of determination. Variabilities of measured exponents are found by bootstrapping over points and deriving slopes by the linear and the Jackknife models [92].

Figure 5 (a) confirms the independence of 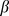 and 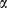 diversity as hypothesized and commonly found in literature [69], [70], [72], [71], [73], [20]. In the specific case of WCA 1, the dissimilarity in species-richness between communities (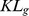) slightly decreases when increasing the local species-richness of communities (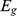). Such trend is more markedly observed when speciation is high and dispersal very limited. In the case of WCA 1 we believe that speciation is fairly high and dispersal is moderately high, however further species sampling are needed to confirm this supposition. This independence between 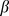 and 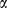 diversity is far to be universal among wetland ecosystems and it is one of the most discussed biodiversity patterns in ecosystems at large. Figure 5 (b) shows that the KL divergence (Equation 4) differs from the difference of Shannon entropies for the green band. In Figure 5 (b) we test the hypothesis of the KL divergence as a better measure of 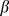 diversity than the difference of Shannon entropies independently of the season considered. The difference of Shannon entropies captures 85% of the 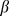 diversity at the most. Instead the KL divergence represents faithfully the whole 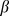 diversity that is estimated on the data. This suggests that interactions and non-linearities in vegetation processes are not negligible. The KL divergence includes the “reciprocal information” of communities in its estimation (Equation 6). Species-turnover can be in fact, lower or higher, than the simple difference of species-richness between the same local communities in time. There may be cases where the disappearance of one species can bring to a large dominance of another species (e.g. this is the case of sawgrass and cattail in many water conservation areas [52]), and cases of “colonization boom” of many alien species that bring the ecosystem to be very species rich (e.g. this is the case of the Everglades National Park [89], [90], [1]). Considering that the reflectance is characterizing species (or functional species) we observe fluctuations of the species richness in time without anomalous spikes that may be related to a net decrease or increase of species. Thus, we can claim that in WCA 1 there have not been relevant issues with alien species as confirmed in [1]. This is potentially related to the confinement of species within WCA 1 by the set of surrounding levees that block immigration of species from outside. Because we did not perform any species classification our conclusion is about average ecosystem properties (in terms of species richness) rather than certainty about no invasion. Some species whose reflectance is similar to endemic species may be introduced in WCA 1 but their number is certain low.

By considering Figure 5 (b), we see that the difference of Shannon entropies is actually a fairly good estimate of 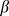 diversity. This may be related to the quite strong time-invariance of species composition for the representative regions chosen for the analysis (Figure 1). Scale dependence in species-turnover has been recently suggested to reflect variance in species occupancy [91]. Thus, the variability of scale of the red squares analyzed might be a source of variability in the estimation of 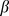 diversity. The similarity between the KL divergence, the difference of Shannon entropies, and 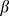 diversity appears moderately high for the dry season. This potentially occurs because of the high spatial heterogeneity in species composition and low reflectance of vegetation during the dry season with respect the wet season. During the dry season the difference in entropies is capable to reproduce only 47% of the observed 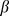 diversity (Figure S6, b). During the wet season the difference in entropies is capable to reproduce 76% of the observed 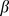 diversity (Figure S6, a). The KL divergence is capable to estimate 61% and 82% of the observed 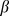 diversity in the dry and in the wet season respectively. Thus, it is advisable to compute the KL divergence rather the difference of Shannon entropies for estimating the 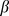 diversity. This is observed to be true both for the interannual and interseasonal species turnover. Overall we verify the superiority of second-oder indices of richness (i.e. the KL divergence) vs first oder indices (i.e. the difference of Shannon entropies) in reproducing 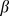 diversity.

**Figure 6:**
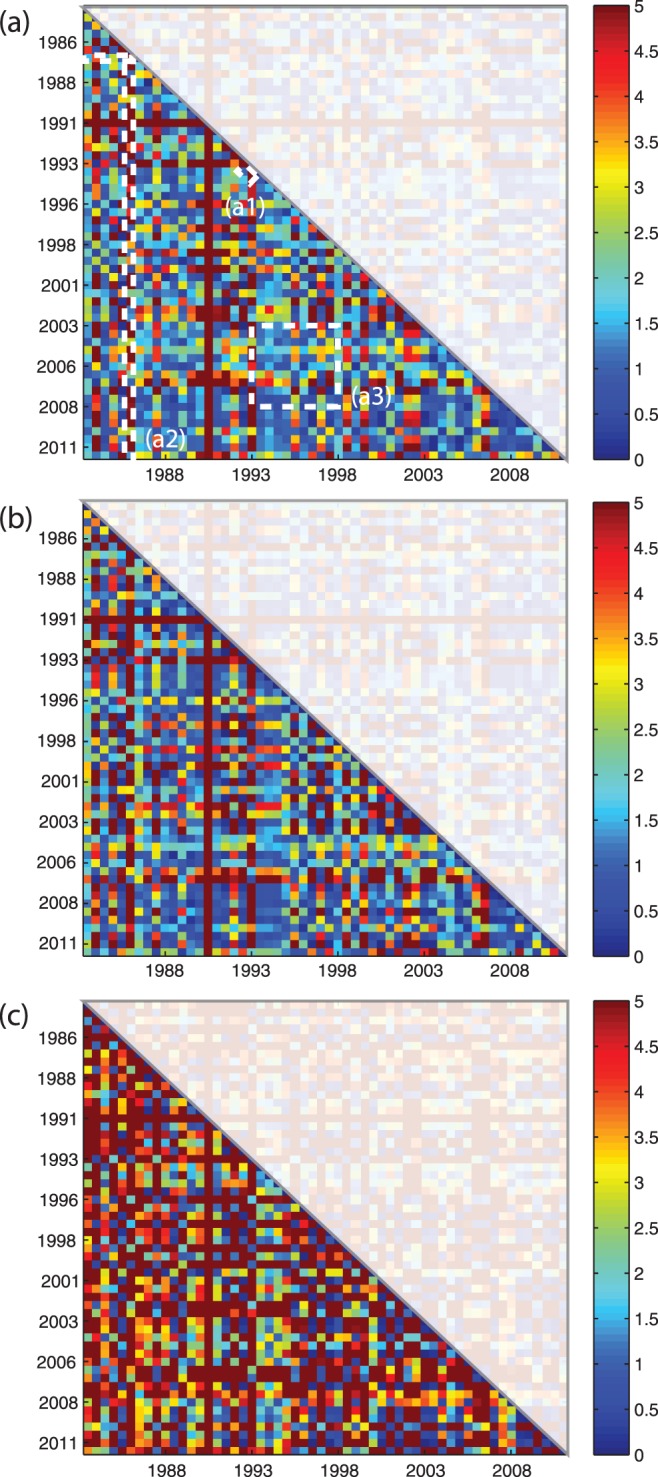
Interseasonal KL divergence matrices for the period 1984–2011. (a), (b), and (c) are the season-season comparisons (56 wet and dry seasons for the period 1984–2011) for the red, green, and blue image band respectively. The more blue/red the pixel the lower/higher the potential 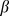 diversity between seasons. Matrices are symmetric and the upper triangular part is made transparent.

In Figure 6 the KL divergence (Equation 4) is represented as a symmetric matrix in which the 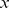 and 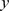 axis are the consecutive seasons (dry and wet) for each year considered in the analysis. The plots are matrices representing the pairwise textural divergence. Figure S7 and S8 provide the KL divergence for the RGB bands for the dry and wet season respectively. The color scale is proportional to the relative generalized Gaussian pairwise distance (i.e. the KL divergence) between images for different seasons/years. The texture is the set of estimated pdfs for each subband, and the dissimilarity measure between textures is based on the KL divergence which is defined between two pdfs (Equation 4). The dissimilarity between and among years is higher for the blue band (Figure 6, c) than for the red and green bands (Figure 6, a and b respectively). In 1991 a red vertical and red horizontal line occurs in the three plots (a, b, and c). Considering the Landsat image in 1991 (Figure 1) we verify that this occurs because of the high cloud cover in that year that makes the analysis unreliable. Thus, this year should be removed by the time series when analyzing temporal variations of 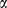 and 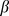 diversity. We include the 1991 image to show that these pairwise textural divergence tables can also be used as a-posteriori indicators to detect images to be excluded in the analysis. The matrices of Figure 6 are useful for comparison of species-richness variation (species-turnover): (i) between seasons of the same or different years (pixels of the upper and lower diagonal along the main diagonal; for example considering the pixels in (a1)); of a selected year with respect to a historical period or with respect to a period in the past (pixels along a column; e.g. considering pixels in (a2)); and, (iii) between periods in time (any group of pixels within a submatrix defined within the matrix; e.g. considering pixels in (a3)). Considering (a1) for example, by taking year 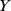 as a row and year 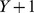 as a column, we are capable to estimate the difference in species between these two years which is the species turnover. Figure S9 reports the KL divergence for the maximum of the RGB signal in the twenty-eight years period considered. Because the maximum KL divergence can be from the red, green, or blue band in this case the KL divergence is representing the overall maximum ecosystem change (being change in vegetation, soil, or water structure) in the period of observation. Thus, this matrix may be useful to detect the periods of maximum change of the ecosystem.

## Discussion

The following points are worth reiterating and discussing.

- The study emphasizes the role of spatial and temporal heterogeneity of the landscape in shaping biodiversity patterns, as a texture method quantifies the strong differences of each season in term of species composition, soil structure and water distribution. Specifically, a positive correlation is found between the Shannon entropy of the red, green, and blue band for the dry and wet seasons and the average annual rainfall of each season. This proves the strong correlation between hydro-geomorphological and ecological changes of wetland ecosystems. The hydro-geomorphological and the ecological changes are represented by the Shannon entropy of the blu-red bands, and of the green band respectively. A negative correlation is found between the RGB band variations of the dry and the wet season as expected because of the opposite ecohydrological dynamics of the two seasons. The texture method quantifies the strong differences of each season in term of species composition, soil structure and water distribution. We believe that our good predictions of species richness from data proves that reflectance is a good metric for assessing taxonomic diversity rather than functional diversity.
- The average species-richness of WCA 1 is found to be constant in the observed period (1984–2011). Despite the decrease in the average annual rainfall from 2000 to present the average species-richness seems to be fairly invariant as reported by [86] and as shown by the data extracted from the Global Biodiversity Information Facility [33]. However, a decrease in species-richness may occur if the drying trend persists in the future. Thus, the monitoring of wetland spectral heterogeneity, performed for example by analyzing image texture, is suggested as a powerful tool in the management of WCA 1 and in general of ecosystems at large. Large-scale restoration requires ecosystem performance measures that can function as rapid quantitative benchmarks of recovery or degradation over time. One of these measures can be the species richness for instance. Our study provides a methodology that can be used for this purpose versus expensive monitoring campaigns post restoration. Our method can also be useful to detect species variation due to species invasion. A novel matrix visualization is proposed to compare the change in species differences (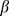 diversity) between seasons, years, and multiple years. This is potentially useful in the analysis of ecological processes and their coupling with anthropic and natural stressors. We also emphasize the utility of fast and accurate multispectral image analysis for ecosystem management versus costly field campaigns as suggested by [18].
- The Shannon entropy and the KL divergence of the green band of satellite imagery are verified as good estimators of the species-richness (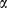 diversity) and of the species turnover (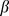 diversity). This is certainly true in ecosystems where spectral heterogeneity is very large. However, the Shannon entropy needs to be calibrated on data in order to assess the number of species as already evidenced by [30]. Our texture analysis is conducted on representative regions of variable extent of the ecosystem analyzed. We found that the predicted 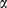 diversity of each representative region is a good proxy the average 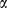 diversity for the whole ecosystem. Moreover we realized that the average 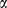 diversity is a lower but close estimate of 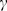 diversity for the ecosystem analyzed after validation of predictions with the data of the Global Biodiversity Information Facility. This proves the scalability of the texture-based analysis method in assessing species richness for ecosystems where species is evenly distributed. For WCA 1 the Shannon entropy of the green band reproduces at least 70% of the observed 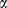 diversity. The Shannon entropy of the green band has a very high cross-correlation with the average annual rainfall for a lag equal to zero. Thus, every fluctuation of the average annual rainfall (every six-months) implies changes in species-richness and community composition. Therefore we believe that the Shannon entropy of the green band can be considered as the spectral heterogeneity (without considering all the bands as in [11]) for assessing 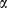 diversity. We verify the assumption of independence of 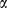 and 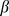 diversity.
- The KL divergence reproduces 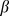 diversity in time (species-turnover) better than the difference of the Shannon entropy of the green band of images between seasons and years. This is because the KL divergence is capable to consider the non-independent variation of species-richness between pairs of local communities which can arise from ecological interactions and feedbacks of local communities. This was proven for 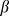 diversity in space by [19]. This is particularly true in the wet season because the higher homogeneity in ecohydrological conditions compared to the dry season makes lower the uncertainty in the estimation of the species-richness. Considering dry and wet seasons together from 1984 to 2011 the difference in Shannon entropy and the KL divergence reproduce 85% and 100% of the observed 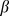 diversity. The KL divergence reproduces 61% and 82% of the observed 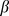 diversity for the dry and wet season respectively. In general our texture analysis method performs better in predicting species turnover than other indicators used in previous studies, for example in [10]. However studies of species turnover are still lacking. We also show that medium spatial resolution Landsat images can provide extremely good estimates of species richness and turnover without the necessity of hyperspectral images.

### Conclusions

We analyze species richness, species turnover, and other spectral heterogeneities derived from satellite imagery for a constructed wetland ecosystem. We use wavelet-based statistical multiresolution texture analysis, a method that, to the best of our knowledge, is new to the field. This method accurately estimates parameters for the marginal distribution of wavelet coefficients using Generalized Gaussian density (GGD) and provides a closed form expression form for the KL divergence between GGD models of different textures. We demonstrate the method on remotely sensed images of the Water Conservation Area 1 in the Greater Everglades Ecosystem Restoration in South Florida but it can applied to any ecosystem. The results suggest that the method is indeed promising for the analysis of species-richness of any ecosystem with high spatial heterogeneity. The method is shown to provide better estimates of 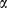 and 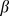 diversity in ecosystems where data-sampling is not feasible (e.g. inaccessible regions) or when it is partially available in time. In particular, statistical wavelet based multiresolution analysis provides a means for estimating divergence between two textures, specifically the Kullback-Leibler divergence between the pair of density functions representing the textures. The KL divergence proved to be a near perfect predictor (*R* ^2^ = 0.98) of species turnover, or 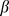 diversity. Additionally, the visualization representing the KL divergence results between texture pairs provide quick insight into species turnover across many years and seasons. It also enables quick recognition of anomalous data. Texture modeling is also helpful to the theoretical understanding of fundamental ecosystem processes, classification of land-cover, classification of species [39], and as inputs to ecohydrological models that are capable of predicting hydroperiod and runoff of wetlands. We anticipate further effort in using Generalized Gaussian, as well as other non-Gaussian multiresolution texture methods, together with the KL and other divergences for comparing textures, in order to achieve several tasks of importance, more accurately at a higher spatiotemporal resolution. These tasks include detecting single species occurrence and abundance through segmentation analysis citref [76], assessing species-dissimilarity in space, and more generally, analyzing satellite imagery including stereoscopic images. Mathematical features for representing textures other than wavelet coefficients and their distribution could also be of interest, especially if they provide models that are simultaneously simple and parsimonous. Moreover, the application of the presented model to other biological systems (for example for the identification of cell communities [44], [76]) and other scales of analysis of ecosystems is a promising research direction. Finally, the methods reduce the need for field-work, while enabling more effective, less costly monitoring, inference, and decision making support.

## Supporting Information

Figure S1 **Remote-sensed images for the Arthur R. Marshall Loxahatchee National Wildlife Refuge (WCA-1) during the wet-season for the period 1987–2011.** The first three years (1984–1986) images are not represented. The representative region in which the texture analysis is performed is delineated in red for each image. The red regions are characterized by a cloud cover lower than 20%. The green regions identify where the data of species are available.
(PDF)

Figure S2 **Interseasonal entropy of WCA-1 Landsat images for the red, green, and blue bands.** (a, b) are the Shannon entropy and average annual rainfall (m) in the period 1984–2011 respectively.
(PDF)

Figure S3
**Cross-correlation between the average annual rainfall and the Shannon entropy of the green-band.** A lag is equivalent to a year. For lag = 0 there is an almost perfect correlation (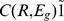) between rainfall and potential 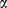 diversity that shows an almost immediate feedback between rainfall and vegetation seasonality.(PDF)

Figure S4
**Predicted 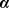 diversity as a function of rainfall.** (a) Shannon entropy for the green band vs. average annual rainfall (m). The maximum of the rainfall is about 600 and 1100 mm in the dry and in the wet season respectively. (b) Shannon entropy for the green band in the dry season vs. average annual rainfall (mm) in the dry season; and, (c) Shannon entropy for the green band in the wet season vs. average annual rainfall (mm) in the wet season. Variabilities of measured exponents are found by bootstrapping over points and deriving slopes by the linear and the Jackknife models [92].(PDF)

Figure S5 **Functional relationships between Shannon entropies of ecosystems components (soil, vegetation, and water spectral signatures).** (a) Shannon entropy for green band vs. blue band, and (b) Shannon entropy for the green band vs red band. The entropy is calculated for every dry and wet season of each year in the period 1984–2011. These relationships hold also considering separately the entropy for the wet- and for the dry season. The dashed grey curves are the 95% confidence interval of the linear regression exponent. Variabilities of measured exponents are found by bootstrapping over points and deriving slopes by the linear and the Jackknife models [92].
(PDF)

Figure S6
**Estimation of the interannual 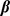 diversity using KL divergence and the difference of Shannon entropies.** Relationship between the interannual KL divergence and the green-band Shannon entropy variation vs. the 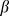 diversity for the period 1984–2011 for the dry and wet seasons respectively (a, and b). The KL divergence better predicts 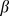 diversity than the difference of the Shannon entropy between years. Variabilities of measured exponents are found by bootstrapping over points and deriving slopes by the linear and the Jackknife models [92].(PDF)

Figure S7
**Interannual KL divergence matrices for the decomposed RGB signal.** Plot (a), (b), and (c) is for the red, green, and blue band, respectively, in the dry season from 1984 to 2011. The higher the KL divergence the higher the dissimilarity (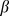 diversity for the green band) between seasons of the same or different years. Matrices are symmetric and the upper triangular part is made transparent.(PDF)

Figure S8
**Interannual KL divergence matrices for the decomposed RGB signal.** Plot (a), (b), and (c) is for the red, green, and blue band, respectively, in the wet season from 1984 to 2011. The higher the KL divergence the higher the dissimilarity (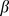 diversity for the green band) between seasons of the same or different years. Matrices are symmetric and the upper triangular part is made transparent.(PDF)

Figure S9 **Interseasonal KL divergence matrix for the maximum of the RGB signal from 1984 to 2011.** The maximum value of the KL divergence can be considered as total ecosystem change (in terms of soil, vegetation, and water) among the years considered. The matrix is symmetric and the upper triangular part is made transparent.
(PDF)

Text S1 **Supporting Study Area Information.**
(PDF)

## References

[1] McVoy C, Said W, Obeysekera J, VanArman J, Dreschel T, editors (2011) Landscape and Hydrology of the Predrainage Everglades. University Press of Florida.

[2] Adler PB, Levine JM (2007) Contrasting relationships between precipitation and species richness in space and time. Oikos 116: 221–232.

[3] Todd M, Muneepeerakul R, Pumo D, Azaele S, Miralles-Wilhelm F, et al (2010) Hydrological drivers of wetland vegetation community distribution within everglades national park, orida. Advances in Water Resources 33: 1279–1289.

[4] Todd MJ, Muneepeerakul R, Miralles-Wilhelm F, Rinaldo A, Rodriguez-Iturbe I (2011) Possible climate change impacts on the hydrological and vegetative character of everglades national park, orida. Ecohydrology.

[5] Cohen MJ, Watts DL, Heffernan JB, Osborne TZ (2011) Reciprocal biotic control on hydrology, nutrient gradients, and landform in the greater everglades. Critical Reviews in Environmental Science and Technology 41: 395–429.

[6] Mitsch WJ, Gosselink JG, editors (2000) Wetlands. John Wiley and Sons, New York.

[7] Bernhardt C, Debra A (2009) Response of the everglades ridge and slough landscape to climate variability and 20th-century water management. Ecological Applications 19: 17231738. 10.1890/08-0779.1 19831066

[8] Prigent C, Matthews E, Aires F, Rossow WB (2001) Remote sensing of global wetland dynamics with multiple satellite data sets. Geophysical Research Letters 28: 4631–4634.

[9] Ferretti M, Chiarucci A (2003) Design concepts adopted in long-term forest monitoring programs in europe-problems for the future? Science of The Total Environment 310: 171–178. 12812741 10.1016/S0048-9697(02)00637-X

[10] Rocchini D, He K, Oldeland J, Wesuls D, Neteler M (2010a) Spectral variation versus species beta-diversity at different spatial scales: a test in african highland savannas. Journal of environmental monitoring JEM 12: 825–831. 20383362 10.1039/b921835a

[11] Rocchini D, Chiarucci A, Loiselle S (2004) Testing the spectral variation hypothesis by using satellite multispectral images. Acta Oecologica 26: 117–120.

[12] Ricotta C, Anand M (2006) Spatial complexity of ecological communities: Bridging the gap between probabilistic and non-probabilistic uncertainty measures. Ecological Modelling 197: 59–66.

[13] Rocchini D, Ricotta C, Chiarucci A (2007b) Using satellite imagery to assess plant species richness: The role of multispectral systems. Applied Vegetation Science 10: 325–331.

[14] Gillespie TW, Foody GM, Rocchini D, Giorgi AP, Saatchi S (2008) Measuring and modelling biodiversity from space. Progress in Physical Geography 32: 203–221.

[15] Nagendra H, Rocchini D, Ghate R, Sharma B, Pareeth S (2010) Assessing Plant Diversity in a Dry Tropical Forest: Comparing the Utility of Landsat and Ikonos Satellite Images. Remote Sensing 2: 478–496.

[16] Southworth J, Munroe D, Nagendra H (2004) Land cover change and landscape fragmentation-comparing the utility of continuous and discrete analyses for a western honduras region. Agriculture, Ecosystems & Environment 101: 185–205.

[17] Belluco E, Camuffo M, Ferrari S, Modenese L, Silvestri S, et al (2006) Mapping saltmarsh vegetation by multispectral and hyperspectral remote sensing. Remote Sensing of Environment 105: 54–67.

[18] Rocchini D, Balkenhol N, Carter G, Foody G, Gillespie T, et al (2010b) Remotely sensed spectral heterogeneity as a proxy of species diversity: Recent advances and open challenges. Ecological Informatics 5: 318–329.

[19] Ludovisi A (2006) TaticchiM (2006) Investigating beta diversity by kullback-leibler information measures. Ecological Modelling 192: 299–313.

[20] Anderson MJ, Crist TO, Chase JM, Vellend M, Inouye BD, et al (2011) Navigating the multiple meanings of beta diversity: a roadmap for the practicing ecologist. Ecology Letters 14: 19–28. 21070562 10.1111/j.1461-0248.2010.01552.x

[21] Buckley LB, Jetz W (2008) Linking global turnover of species and environments. Proceedings of the National Academy of Sciences of the United States of America 105: 17836–17841. 19001274 10.1073/pnas.0803524105 PMC2584760

[22] Lhermitte S, Verbesselt J, Verstraeten W, Coppin P (2011) A comparison of time series similarity measures for classification and change detection of ecosystem dynamics. Remote Sensing of Environment 115: 3129–3152.

[23] Latifi H, Fassnacht F, Koch B (2012) Forest structure modeling with combined airborne hyperspectral and lidar data. Remote Sensing of Environment 121: 10–25.

[24] Poveda G, Salazar L (2004) Annual and interannual (enso) variability of spatial scaling properties of a vegetation index (ndvi) in amazonia. Remote Sensing of Environment 93: 391–401.

[25] Saatchi S, Buermann W, ter Steege H, Mori S, Smith T (2008) Modeling distribution of amazonian tree species and diversity using remote sensing measurements. Remote Sensing of Environment 112: 2000–2017.

[26] Tomppo E, Gagliano C, Natale FD, Katila M, McRoberts R (2009) Predicting categorical forest variables using an improved k-nearest neighbour estimator and landsat imagery. Remote Sensing of Environment 113: 500–517.

[27] SFWMD (2000) Ecological effects of phosphorus enrichment in the everglades. chapter 3. In: Redfield G, editor, Everglades Consolidated Report., South Florida Water Management District.

[28] Cohen M, Grunwald S, Clark M, Reddy K (2007) Rapid assessment of restoration performance measures at multiple scales in the greater everglades using near infrared reectance spectroscopy (nirs). Technical report, Institute of Food and Agricultural Sciences, University of Florida. http://www.nps.gov/ever/naturescience/upload/ASS04-1FinalReportSecure.pdf; Accessed 2012.

[29] Do MN, Vetterli M (2002) Wavelet-based texture retrieval using generalized Gaussian density and kullback-leibler distance. IEEE Trans Image Processing 11: 146–158. 10.1109/83.982822 18244620

[30] Jost L (2006) Entropy and diversity. Oikos 113: 363–375.

[31] Rocchini D (2009) Algorithmic foundation of spectral rarefaction for measuring satellite imagery heterogeneity at multiple spatial scales. Sensors 9: 303–310. 22389600 10.3390/s90100303 PMC3280746

[32] Viedma O, Torres I, Perez B, Moreno J (2012) Modeling plant species richness using reectance and texture data derived from quickbird in a recently burned area of central spain. Remote Sensing of Environment 119: 208–221.

[33] GBIF (2012) Global biodiversity information facility. Technical report, Global Biodiversity Information Facility. http://www.gbif.org/; Accessed 2012.

[34] McGlinn D, Palmer M (2010) Quantifying the inuence of environmental texture on the rate of species turnover: evidence from two habitats. Plant Ecology: 1–12.

[35] Marcon E, Herault B, Baraloto C, Lang G (2012) The decomposition of shannon's entropy and a confidence interval for beta diversity. Oikos 121: 516–522.

[36] St-Louis V, Pidgeon A, Clayton M, Locke B, Bash D, et al (2009) Satellite image texture and a vegetation index predict avian biodiversity in the chihuahuan desert of new mexico. Ecography 32: 468–480.

[37] Bellis L, Pidgeon A, Radeloff V, Navarro J, Martella M (2008) Modeling habitat suitability for the endangered greater rhea (rhea americana) in central argentina based on satellite image texture. Ecological Applications 18: 1956–1966. 19263890 10.1890/07-0243.1

[38] Gallardo-Cruz, Meave JA, González EJ, Lebrija-Trejos EE, Romero-Romero MA, et al.. (2012) Predicting tropical dry forest successional attributes from space: Is the key hidden in image texture? PLoS ONE 7. 10.1371/journal.pone.0030506 PMC3282724 22363443

[39] Key T, Warner TA, McGraw JB, Fajvan MA (2001) A comparison of multispectral and multitemporal information in high spatial resolution imagery for classification of individual tree species in a temperate hardwood forest. Remote Sensing of Environment 75: 100–112.

[40] Townsend AR, Asner GP, Cleveland CC (2008) The biogeochemical heterogeneity of tropical forests. Trends in Ecology & Evolution 23: 424–431. 18582987 10.1016/j.tree.2008.04.009

[41] Wulder M (2004) High spatial resolution remotely sensed data for ecosystem characterization. Biogeoscience 54: 511–521.

[42] Jeffreys C (2004) Support vector machine and parametric wavelet-based texture classification of stem cell images – phd thesis. Technical report, Massachusetts Institute of Technology. http://dspace.mit.edu/bitstream/handle/1721.1/16651/56472941. pdf?sequence = 1; Accessed 2012.

[43] Mangoubi R, Jeffrey C, Copeland A, Desai M, Sammak P (2007) Non-invasive image based support vector machine classification of human embryonic stem cells. International Symposium on Biomedical Imaging: 284–287.

[44] Mangoubi R, Desai M, Sammak P (2008) Non-gaussian methods in biomedical imaging. Applied Image Pattern Recognition Workshop 0: 1–6.

[45] Childers D, Doren R, Jones R, Noe G, Rugge M, et al.. (2003) Decadal change in vegetation and soil phosphorus pattern across the everglades landscape. J Environ Qual 32. 10.2134/jeq2003.3440 12549575

[46] GLOVIS (2011) Global visualization viewer. Technical report, USGS. http://glovis.usgs.gov/; Accessed 2012.

[47] USGS (2011) Earth explorer. Technical report, USGS. http://edcsns17.cr.usgs.gov/NewEarthExplorer/; Accessed 2012.

[48] USGS (2003) Preliminary assessment of the value of landsat 7 etm+ data following scan line corrector malfunction. Technical report, U.S. Geological Survey, EROS Data Center, NASA, and Landsat 7 Science Team. http://landsat.usgs.gov/documents/SLC_off_Scientific_Usability.pdf; Accessed 2012.

[49] USACE (2012) Comprehensive everglades restoration project database. Technical report, US Army Corps of Engineers. http://www.cerpzone.org/; Accessed 2012.

[50] Convertino M, Muneepeerakul R, Azaele S, Bertuzzo E, Rinaldo A, et al (2009) On neutral metacommunity patterns of river basins at different scales of aggregation. Water Resources Research 45: 8424.

[51] Azaele S, Cornell S, Kunin W (2012) Downscaling species occupancy from coarse spatial scales. Ecological Applications: in press. 10.1890/11-0536.1 22645828

[52] Lagerwall G, Kiker G, Munoz-Carpena R, Convertino M, James A, et al.. (2012) A spatially- distributed, deterministic approach to modeling typha domingensis (cattail) in an everglades water- controlled wetland. Ecological Processes; special issue “Wetland in a Complex World” forthcoming.

[53] Cho HJ (2007) Depth-variant spectral characteristics of submersed aquatic vegetation detected by landsat 7 etm+. Int J Remote Sens 28: 1455–1467.

[54] Nagendra H, Rocchini D (2008) High resolution satellite imagery for tropical biodiversity studies: the devil is in the detail. Biodiversity and Conservation 17: 3431–3442.

[55] Rose P, Rosendahl P (1979) An application of landsat multispectral imagery for the classification of hydrobiological systems, shark river slough, everglades national park, orida. Technical report, South Florida Research Center Report. http://www.nps.gov/ever/naturescience/upload/SecureTRT-544.pdf; Accessed 2012.

[56] Bubier JL, Rock BN, Crill PM (1997) Spectral reectance measurements of boreal wetland and forest mosses. Journal of Geophysical Research 102.

[57] Rundquist D, Narumalani S, Narayanan R (2001) A review of wetlands remote sensing and defining new considerations. Remote Sensing Reviews 20: 207–226.

[58] Ozesmi SL, Bauer ME (2002) Satellite remote sensing of wetlands. Wetlands Ecology and Management 10: 381402.

[59] Petchey OL, Gaston KJ (2002) Functional diversity (FD), species richness and community composition. Ecology Letters 5: 402–411.

[60] Tilley D, Baldwin A, Jenkins E (2004) Leaf-scale hyperspectral reectance models for determining the nitrogen status of freshwater wetlands. Technical report, Maryland Water Resources Research Center.

[61] Rocchini D (2007a) Effects of spatial and spectral resolution in estimating ecosystem *α*-diversity by satellite imagery. Remote Sensing of Environment 111: 423–434.

[62] Wang C, Menenti M, Stoll MP, Belluco E, Marani M (2007) Mapping mixed vegetation communities in salt marshes using airborne spectral data. Remote Sensing of Environment: 559–570.

[63] Ajithkumar TT, Thangaradjou T, Kannan L (2008) Spectral reectance properties of mangrove species of the muthupettai mangrove environment, tamil nadu. Journal of Environmental Biology 29: 785–78. 19295083

[64] Sun Y, Liu XH, Wu Y (2011) Identifying hyperspectral characteristics of wetland species using in situ data. ISPRS Journal of Photogrammetry and Remote Sensing.

[65] Miao S, Zou CB (2009) Seasonal variation in seed bank composition and its interaction with nutrient enrichment in the everglades wetlands. Aquatic Botany 90: 157–164.

[66] Rocchini D, Neteler M (2012) Spectral rank-abundance for measuring landscape diversity. International Journal of Remote Sensing 33: 4458–4470.

[67] Thenkabail PS, Enclona EA, Ashton MS, Meer BVD (2004) Accuracy assessments of hyperspectral waveband performance for vegetation analysis applications. Remote Sensing of Environment 91: 354–376.

[68] Muneepeerakul R, Rinaldo A, Levin SA, Rodriguez-Iturbe I (2008) Signatures of vegetational functional diversity in river basins. Water Resources Research 44.

[69] Jost L (2007) Partitioning diversity into independent alpha and beta components. Ecology 88: 2427–2439. 18027744 10.1890/06-1736.1

[70] Baselga A (2010) Multiplicative partition of true diversity yields independent alpha and beta components; additive partition does not. Ecology 91: 1974–1981. 20715618 10.1890/09-0320.1

[71] Jost L (2010) Independence of alpha and beta diversities. Ecology 91: 1969–1974. 20715617 10.1890/09-0368.1

[72] Veech JA, Crist TO (2010) Diversity partitioning without statistical independence of alpha and beta. Ecology 91: 1964–1969. 20715616 10.1890/08-1727.1

[73] Wilsey BJ (2010) An empirical comparison of beta diversity indices in establishing prairies. Ecology 91: 1984–1988. 20715620 10.1890/09-0351.1

[74] Brandt L, KitchensW(1998) Spatial and temporal changes in tree islands of the arthur r. marshall loxahatchee national wildlife refuge in response to altered hydrologies. Technical report, Florida Cooperative Fish and Wildlife Research Unit, University of Florida.

[75] Engler O, Randle V, editors (2009) Introduction to Texture Analysis: Macrotexture, Microtexture, and Orientation Mapping. CRC Press.

[76] Lowry N, Desai M, Mangoubi R, Sammak P (2010) Nonparametric segmentation and classification of small size irregularly shaped stem cell nuclei using adjustable windowing. IEEE International Symposium on Biomedical Imaging: From Nano to Macro: 141–144.

[77] Erb T, Schneider C, Mucko S, Sanfilippo J, Lowry N, et al (2011) Paracrine and epigenetic control of trophectoderm differentiation from human embryonic stem cells: the role of bone morphogenic protein 4 and histone deacetylases. Stem Cells Dev 20: 1601–14. 21204619 10.1089/scd.2010.0281 PMC3161106

[78] Van DeWouwer G, Scheunders P, Van Dyck D (1999) Statistical texture characterization from discrete wavelet representations. IEEE Transactions on Image Processing 8: 592–598. 18262903 10.1109/83.753747

[79] Mangoubi R, Desai M, Sammak P (2008) Performance evaluation of multiresolution texture analysis of stem cell chromatin. IEEE International Symposium on Biomedical Imaging: From Nano to Macro: 380–383.

[80] MaCarthur R (1965) Patterns of species diversity. Biological Reviews 40: 510–533.

[81] Whittaker RH (1960) Vegetation of the siskiyou mountains, oregon and california. Ecological Monographs 30.

[82] Hill MO (1973) Diversity and evenness: a unifying notation and its consequences. Ecology 54: 427–432.

[83] Keylock CJ (2005) Simpson diversity and the shannonwiener index as special cases of a generalized entropy. Oikos 109: 203–207.

[84] Connolly J, Cadotte M, Brophy C, Dooley A, Finn J, et al (2011) Phylogenetically diverse grasslands are associated with pairwise interspecific processes that increase biomass. Ecology 92: 13851392. 10.1890/10-2270.1 21870611

[85] Kullback S, Leibler R (1951) On information and sufficiency. Ann Math Stat 22: 79–86.

[86] Childers D, Boyer J, Davis S, Madden C, Rudnick D, et al (2006) Relating precipitation and water management to nutrient concentrations in the oligotrophic “upside-down” estuaries of the orida everglades. Limnol Oceanogr 51: 602616.

[87] Yesson C, Brewer P, Sutton T, Caithness N, Pahwa J, et al.. (2007) How global is the global biodiversity information facility? PLoS ONE 2. 10.1371/journal.pone.0001124 PMC2043490 17987112

[88] Jurasinski G, Retzer V, Beierkuhnlein C (2009) Inventory, differentiation, and proportional diversity: a consistent terminology for quantifying species diversity. Oecologia 159: 15–26. 18953572 10.1007/s00442-008-1190-z

[89] Daehler CC (2003) Performance comparisons of Co-Occurring native and alien invasive plants: Implications for conservation and restoration. Annual Review of Ecological and Evolutionary Systematics 34.

[90] Zedler JB, Kercher S (2004) Causes and consequences of invasive plants in wetlands: Opportunities, opportunists, and outcomes. Critical Reviews in Plant Sciences 23: 431–452.

[91] McGlinn D, Hurlbert A (2012) Scale dependence in species turnover reects variance in species occupancy. Ecology. 10.1890/11-0229.1 22624311

[92] Warton DI, Wright IJ, Falster DS, Westoby M (2006) Bivariate line-fitting methods for allometry. Biological Reviews 81: 259–291. 16573844 10.1017/S1464793106007007

