# Supplementary figures and images for: Inferring Species Richness and Turnover by Statistical Multiresolution Texture Analysis of Satellite Imagery
Source: PLoS One. 2012 Oct 24;7(10):e46616. doi: 10.1371/journal.pone.0046616 (PMC3480366; doi:10.1371/journal.pone.0046616)

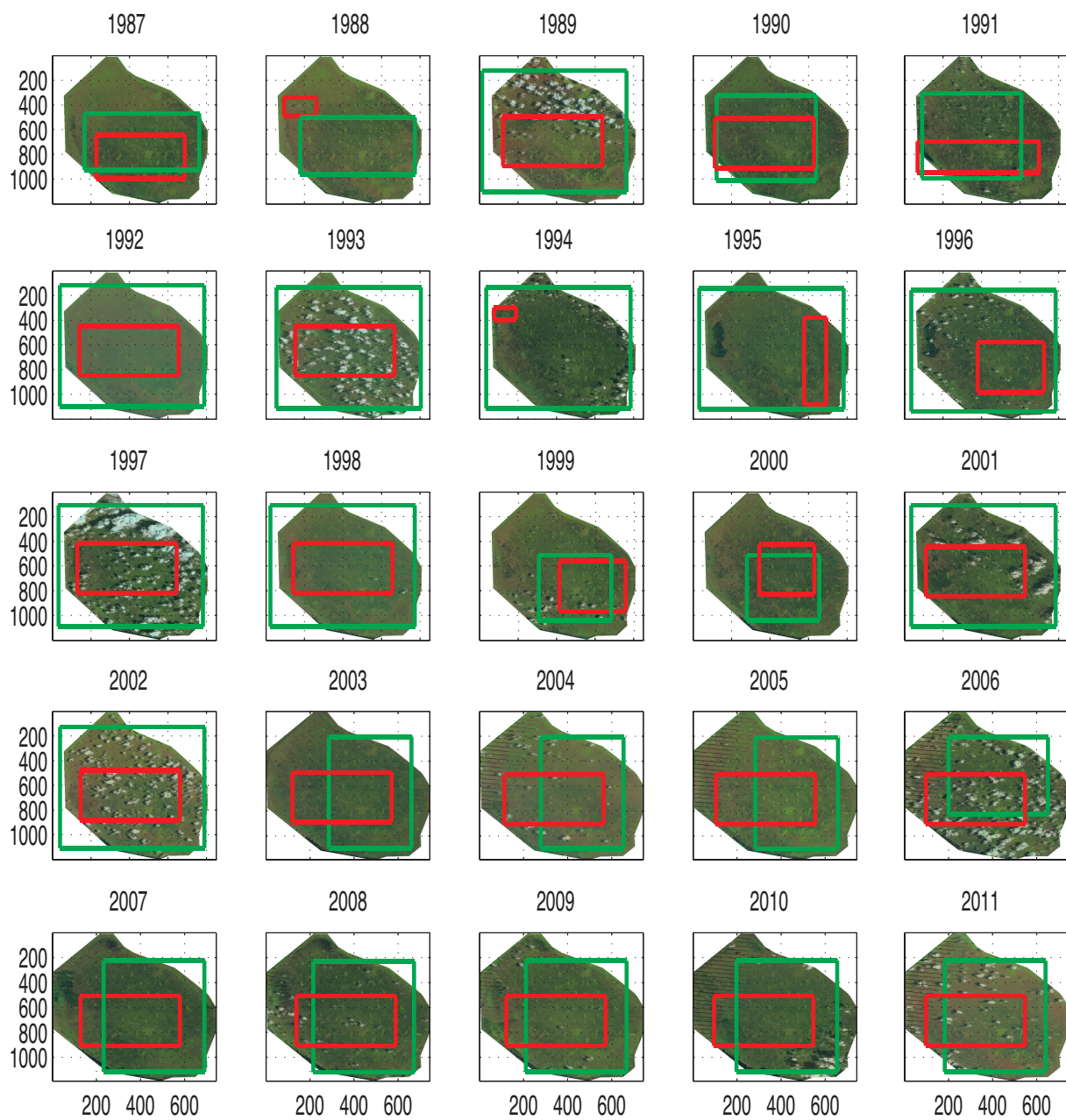

Figure S1:

Supplement: Figure S1 — Remote-sensed images for the Arthur R. Marshall Loxahatchee National Wildlife Refuge (WCA-1) during the wet-season for the period 1987–2011. The first three years (1984–1986) images are not represented. The representative region in which the texture analysis is performed is delineated in red for each image. The red regions are characterized by a cloud cover lower than 20%. The green regions identify where the data of species are available. (PDF) [file pone.0046616.s001.pdf]

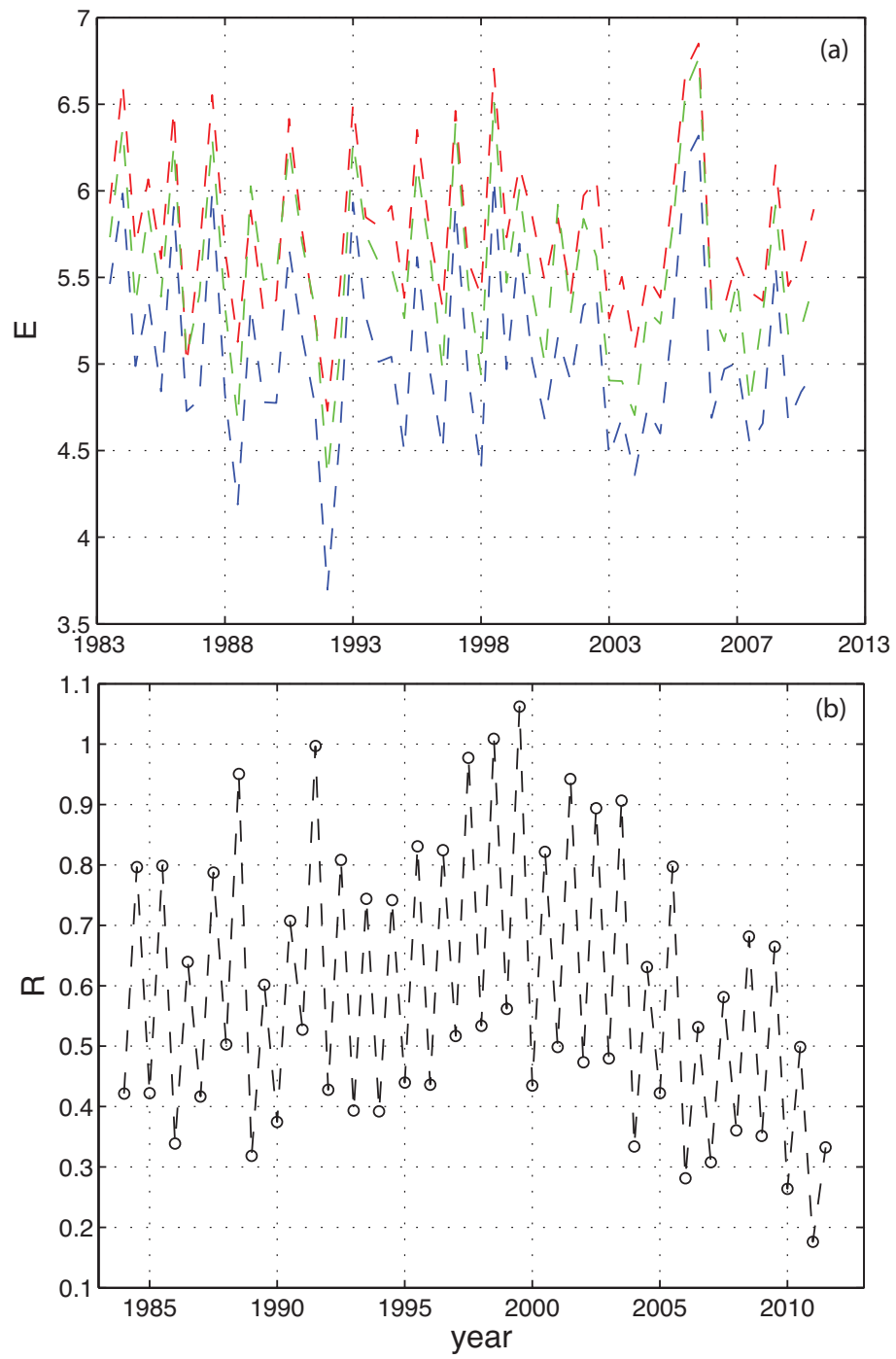

Figure S2:

Supplement: Figure S2 — Interseasonal entropy of WCA-1 Landsat images for the red, green, and blue bands. (a, b) are the Shannon entropy and average annual rainfall (m) in the period 1984–2011 respectively. (PDF) [file pone.0046616.s002.pdf]

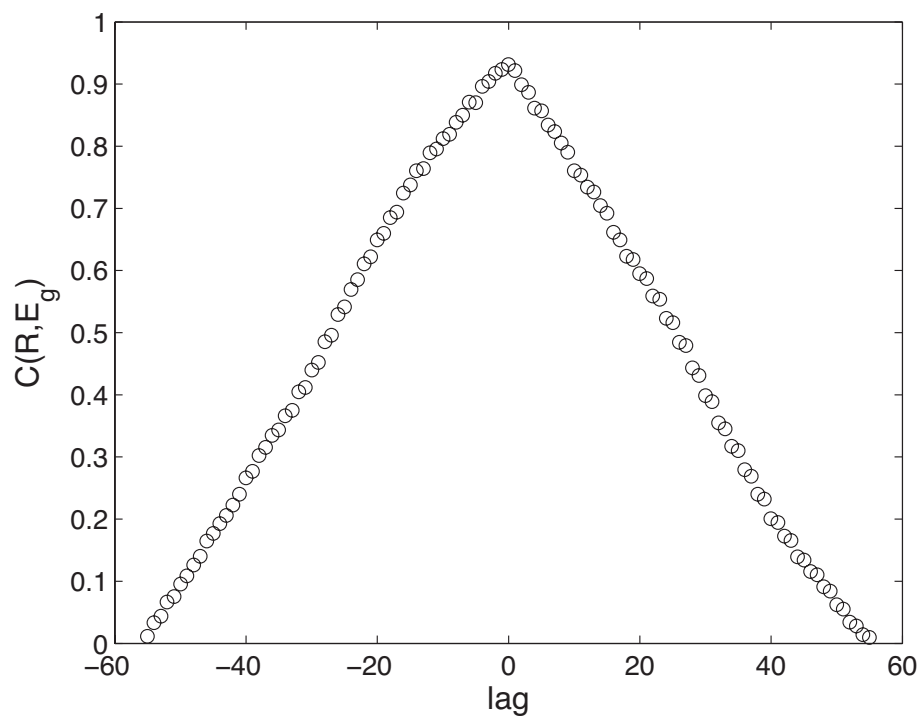

Figure S3:

Supplement: Figure S3 — Cross-correlation between the average annual rainfall and the Shannon entropy of the green-band. A lag is equivalent to a year. For lag = 0 there is an almost perfect correlation () between rainfall and potential diversity that shows an almost immediate feedback between rainfall and vegetation seasonality. (PDF) [file pone.0046616.s003.pdf]

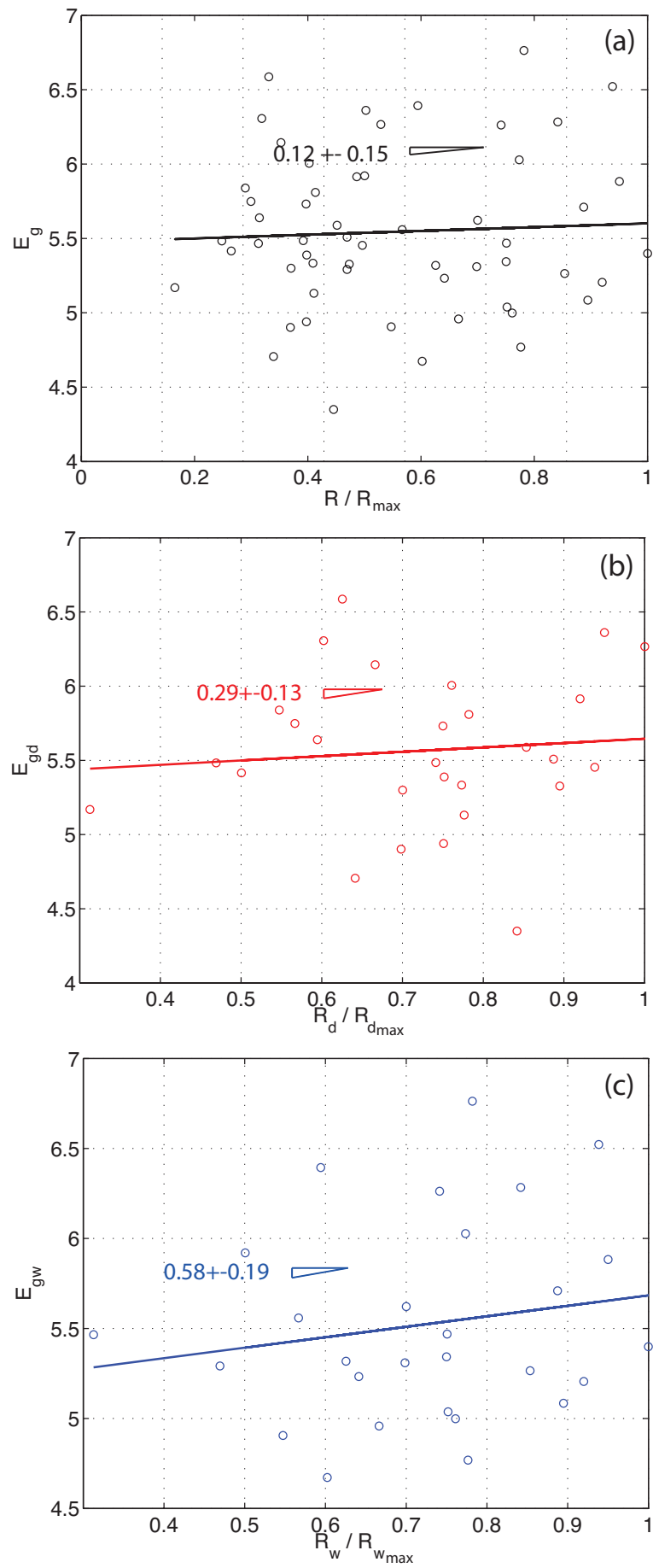

Figure S4:

Supplement: Figure S4 — Predicted diversity as a function of rainfall. (a) Shannon entropy for the green band vs. average annual rainfall (m). The maximum of the rainfall is about 600 and 1100 mm in the dry and in the wet season respectively. (b) Shannon entropy for the green band in the dry season vs. average annual rainfall (mm) in the dry season; and, (c) Shannon entropy for the green band in the wet season vs. average annual rainfall (mm) in the wet season. Variabilities of measured exponents are found by bootstrapping over points and deriving slopes by the linear and the Jackknife models [92]. (PDF) [file pone.0046616.s004.pdf]

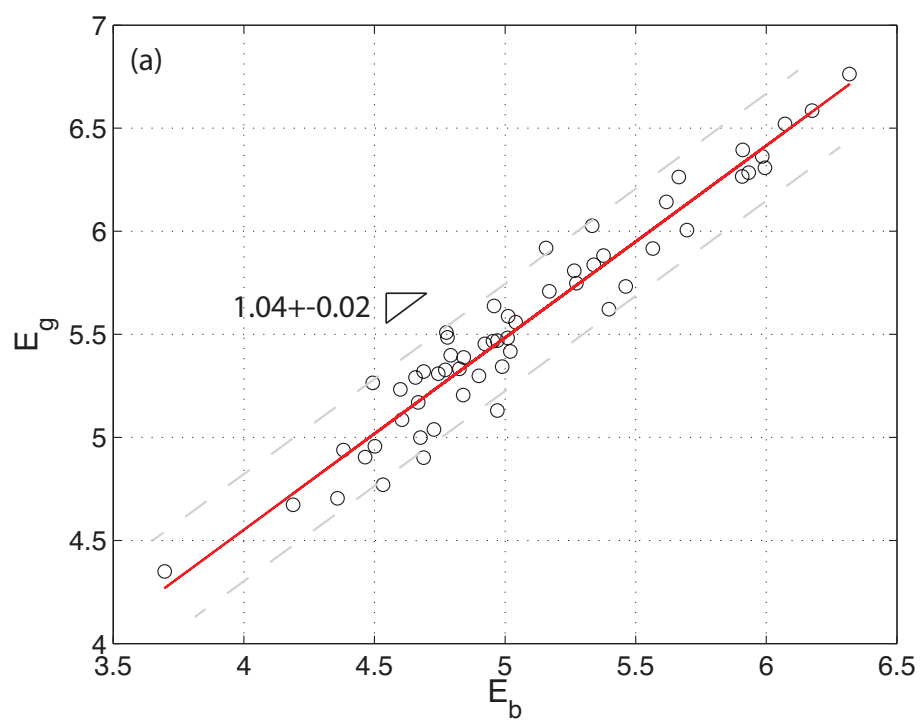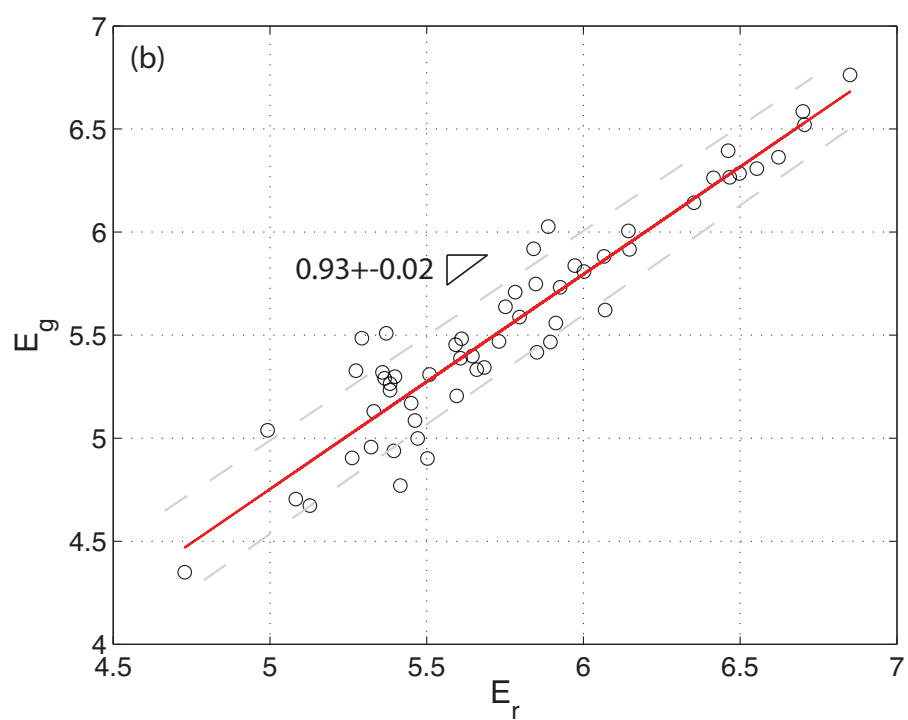

Figure S5:

Supplement: Figure S5 — Functional relationships between Shannon entropies of ecosystems components (soil, vegetation, and water spectral signatures). (a) Shannon entropy for green band vs. blue band, and (b) Shannon entropy for the green band vs red band. The entropy is calculated for every dry and wet season of each year in the period 1984–2011. These relationships hold also considering separately the entropy for the wet- and for the dry season. The dashed grey curves are the 95% confidence interval of the linear regression exponent. Variabilities of measured exponents are found by bootstrapping over points and deriving slopes by the linear and the Jackknife models [92]. (PDF) [file pone.0046616.s005.pdf]

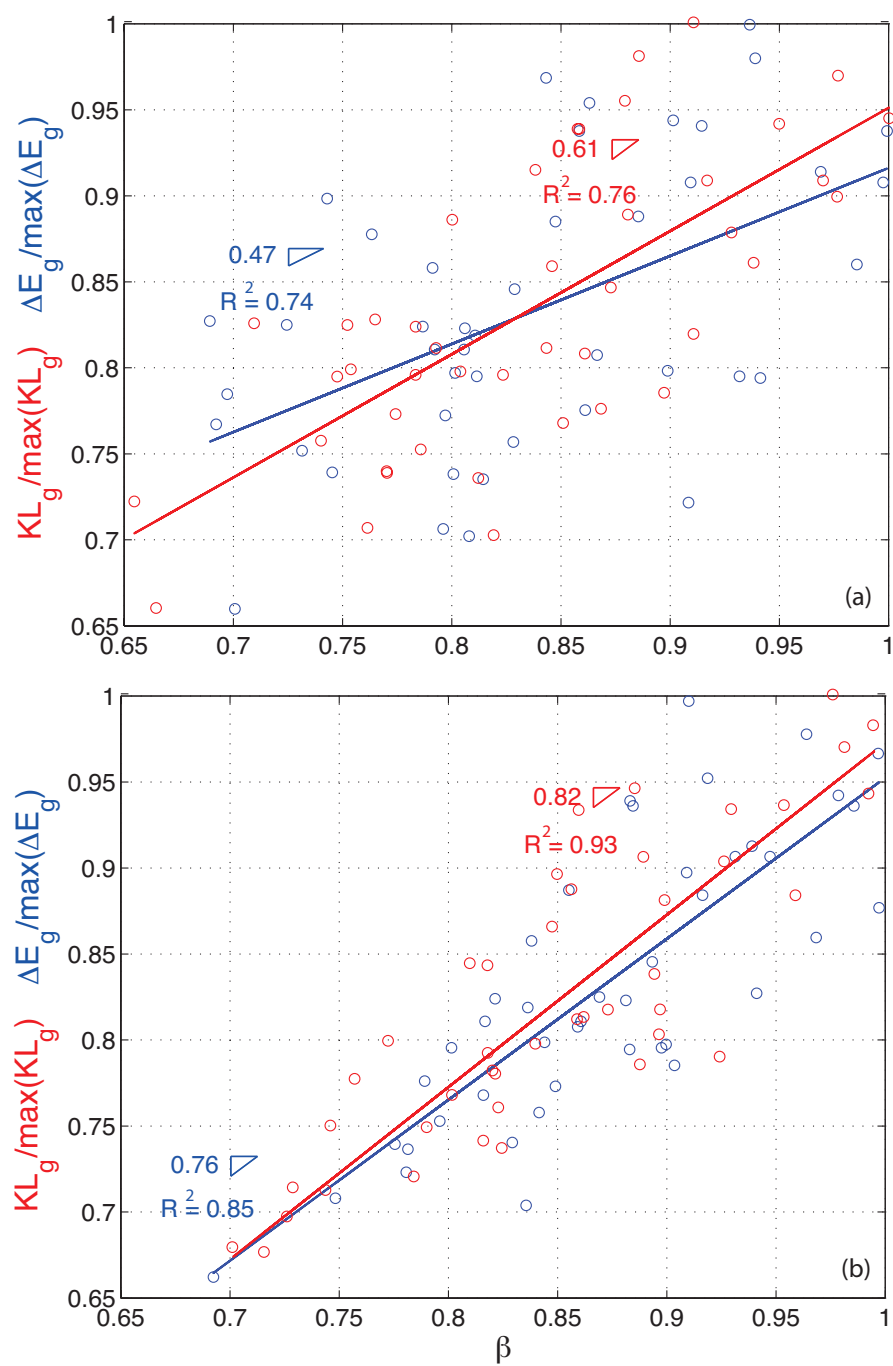

Figure S6:

Supplement: Figure S6 — Estimation of the interannual diversity using KL divergence and the difference of Shannon entropies. Relationship between the interannual KL divergence and the green-band Shannon entropy variation vs. the diversity for the period 1984–2011 for the dry and wet seasons respectively (a, and b). The KL divergence better predicts diversity than the difference of the Shannon entropy between years. Variabilities of measured exponents are found by bootstrapping over points and deriving slopes by the linear and the Jackknife models [92]. (PDF) [file pone.0046616.s006.pdf]

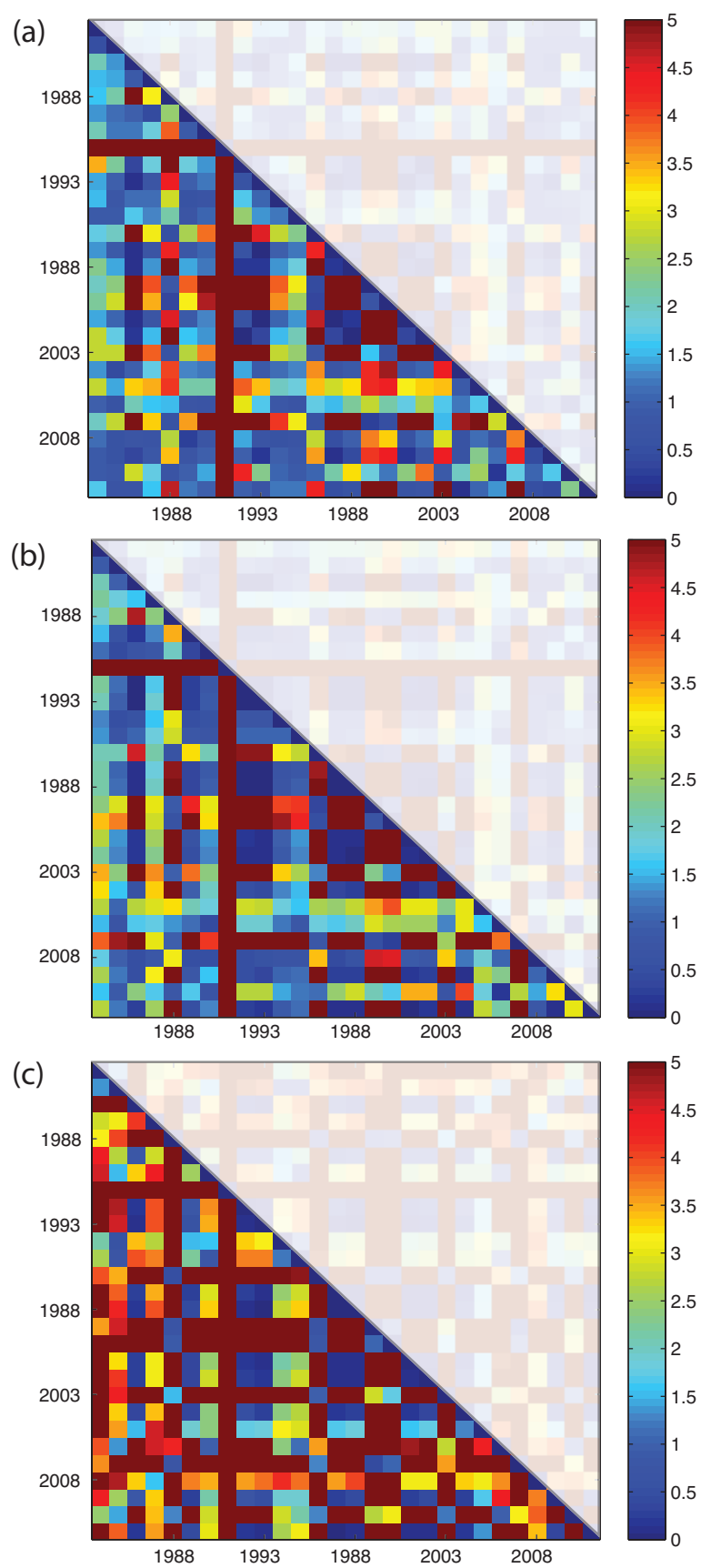

Figure S7:

Supplement: Figure S7 — Interannual KL divergence matrices for the decomposed RGB signal. Plot (a), (b), and (c) is for the red, green, and blue band, respectively, in the dry season from 1984 to 2011. The higher the KL divergence the higher the dissimilarity ( diversity for the green band) between seasons of the same or different years. Matrices are symmetric and the upper triangular part is made transparent. (PDF) [file pone.0046616.s007.pdf]

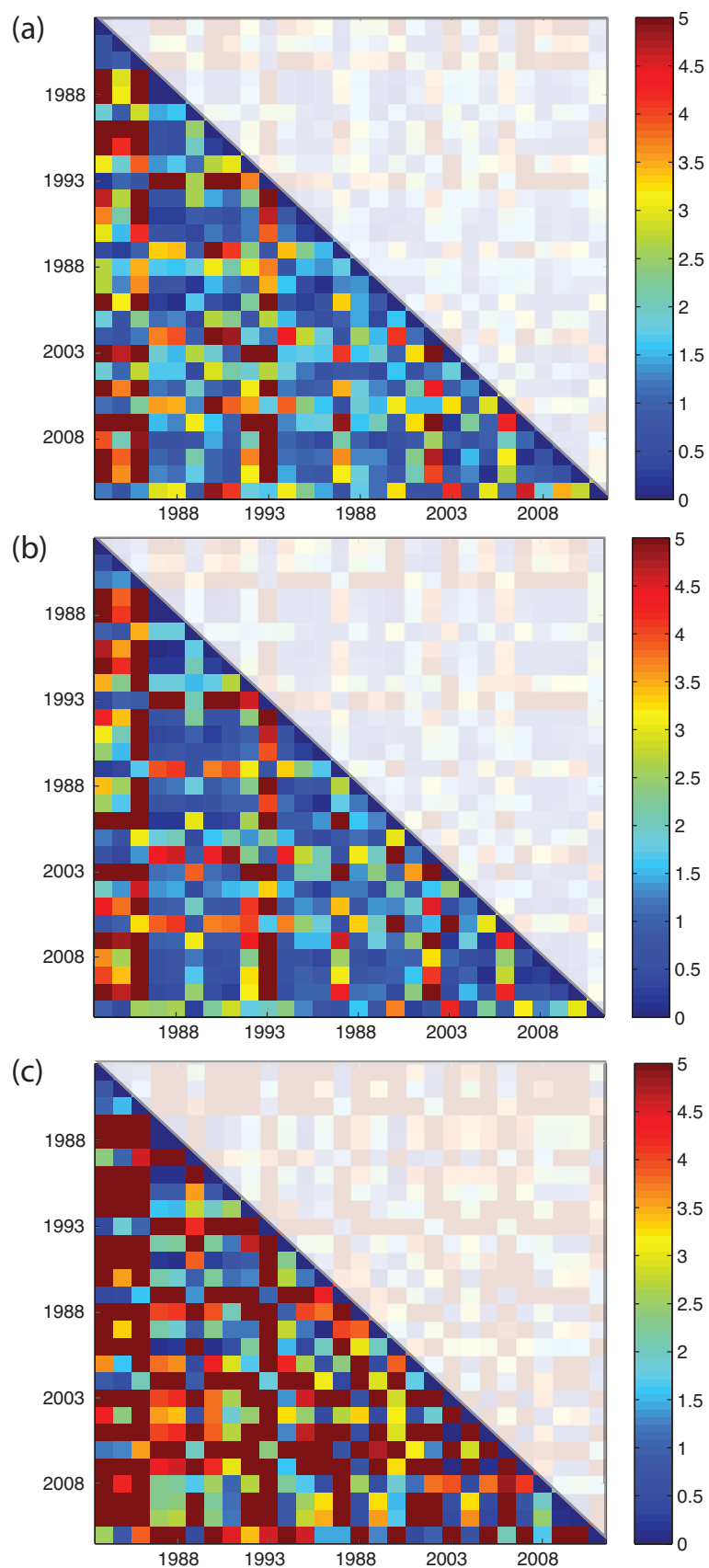

Figure S8:

Supplement: Figure S8 — Interannual KL divergence matrices for the decomposed RGB signal. Plot (a), (b), and (c) is for the red, green, and blue band, respectively, in the wet season from 1984 to 2011. The higher the KL divergence the higher the dissimilarity ( diversity for the green band) between seasons of the same or different years. Matrices are symmetric and the upper triangular part is made transparent. (PDF) [file pone.0046616.s008.pdf]

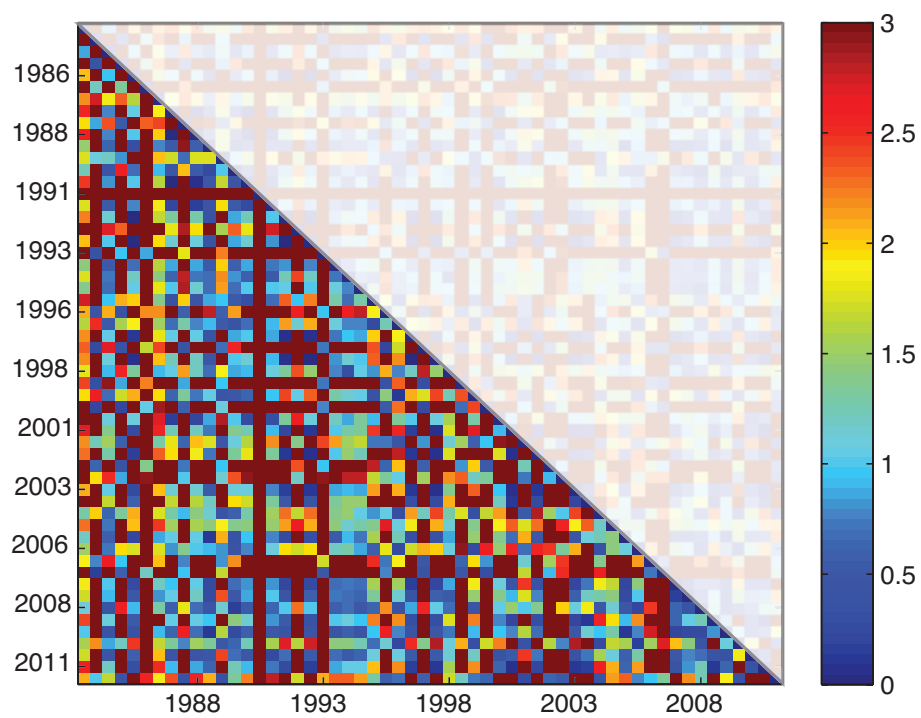

Figure S9:

Supplement: Figure S9 — Interseasonal KL divergence matrix for the maximum of the RGB signal from 1984 to 2011. The maximum value of the KL divergence can be considered as total ecosystem change (in terms of soil, vegetation, and water) among the years considered. The matrix is symmetric and the upper triangular part is made transparent. (PDF) [file pone.0046616.s009.pdf]
