# Supplementary material for: Inferring Species Richness and Turnover by Statistical Multiresolution Texture Analysis of Satellite Imagery
Source: PLoS One. 2012 Oct 24;7(10):e46616. doi: 10.1371/journal.pone.0046616 (PMC3480366; doi:10.1371/journal.pone.0046616)
Supplement: Text S1 — Supporting Study Area Information. (PDF) [file pone.0046616.s010.pdf]

S1. Supporting Study Area Information

Water Conservation Area 1 (WCA 1), also known as the Arthur R. Marshall Loxahatchee National Wildlife Refuge (LNWR) was created by a water management plan (Central and South Florida Project for Flood Control and Other Purposes) that included the construction of levees and canals to create three WCAs from previously drained lands [1, 2]. These three WCAs (1, 2 and 3) provide water supply and storage, flood protection, and environmental benefits to the region. These surrounding levees and spillways of WCA 1 were largely operational by 1961. WCA 1 is comprised of approximately 145,280 acres (588 km^2) of northern Everglades wetlands [2]. Historically, WCA 1 was hydrologically interconnected with Water Conservation Areas 2 and 3, and the Everglades National Park, which together formed the vast overland flow Everglades system that extended from Lake Okeechobee to Florida Bay [2]. Rain was the primary hydrologic source during most of the year, however during wetter periods, overflow from Lake Okeechobee resulted in occasional pulses which followed a natural north to south or northwest to southeast flow pattern. This combination of hydrologic pulses, in conjunction with typical rainfall conditions, helped to create the natural Everglades landscape (ridge and slough) in this area [3]. Because the majority (54 %) of WCA 1's water budget continues to originate from rainfall, WCA 1 exhibits unique background conditions characterized by soft (slightly acidic, low mineral) low nutrient water. In contrast to WCA-2 where mineral rich canal inflows comprise a large portion of the hydrologic input, surface waters within the interior of WCA 1 have extremely low concentrations of major ions such as sodium, calcium, and carbonate [1]. Thus, WCA 1 has a strong correlation between rainfall and ecological dynamics. The climatological pattern of South Florida is characterized by two seasons. The yearly rainfall is characterized by a dry season from December to April, and by a wet season from May to November. During the dry season the vegetation is "dormant" and the green component of the landscape is much less evident. During the wet season that corresponds to the summer activity of tropical cyclones the vegetation shows its maximum productivity. Changes of vegetation activity, composition, and richness due to fire, and nutrients are possible and detectable, however the rainfall is repute to be the main driver of vegetation patterns [2].

The most prevalent vegetation pattern type found in WCA 1 is composed by slough and tree-island areas primarily comprised of open water, aquatic vegetation, and groups of trees. Commonly found slough species included water-lily (*Nymphaea odorata*), floating heart (*Nymphoides aquatica*), and bladderwort (*Utricularia spp.*). The second vegetation pattern type is sawgrass (*Cladium jamaicense*) and wax-myrtle (*Myrica cerifera*) thickets, which forms transitional zones between uniform (monospecific) sawgrass stands and slough/tree-

island areas. The tree islands found within WCA 1 consist of both small, round bayheads and large elongated strand islands ranging in length from several meters to several kilometers and in size from 0.01 to 100 acres. Figure S1 and Figure S1 (Supporting Material) clearly show the tree islands for each year of the analysis for the dry and wet season respectively. We observe an average invariance in time of the landscape patterns at the macroscale. For example, the position and shape of tree-islands is on average the same among years. The time-invariance assumption of landscape patterns particularly holds for WCA 1 among other water conservation areas in the Greater Everglades Ecosystem. As for the scale-invariance, the Everglades ecosystems is highly complex and it is impossible to claim the existence of scale-invariant patterns [4]. For this motivation we try to select regions of analysis that are representative of the whole landscape of WCA 1, and as large as possible.

Man-made alterations to the Everglades ecosystem in the past century resulted in measurable effects on the vegetation of Loxahatchee National Wildlife Refuge. A detailed understanding of these effects is necessary to gauge the extent of previous impacts and the potential for future impacts. It is clear that hydrological modifications to the sheetflow system affecting hydroperiod and water quality had significant impact on present day vegetation in the area. Water timing and delivery has been altered so that some areas have shorter hydroperiods, while others experience extended periods of inundation. In WCA 1, this has resulted in the northern end being overdrained and drier than normal and the southern end being inundated with standing water most of the year [5]. Because of the strong link between the hydrological and ecological dynamics many vegetation changes in plant species-richness have been observed. Particularly, [6] and [7] reported many hydro-ecological changes measured by field measurements that are used in our analysis. For example, since 1970 observations indicate that northern tree islands are losing their distinctive elongated shape [8]. Additionally, pooling water in the south has stressed and drowned out many tree islands. Changes to other habitats are also occurring, with areas having shortened hydroperiods experiencing a shift to woody vegetation, like wax myrtle and willow (*Salix caroliniana*). While areas with increased hydroperiods demonstrate shifts toward aquatic species. An analysis conducted by U.S. Fish and Wildlife Service in the early 1970s found that plant communities at low elevations were experiencing a shift towards aquatic habitats, which resulted in increases in the abundance of several nuisance species including hydrilla (*Hydrilla veticillatum*), water hyacinth (*Eichhornia crasipes*), water lettuce (*Pistia stratiotes*), and cattail (*Typha spp.*) [9]. These changes occurring at the southern end of the Refuge are confounded by the effects of the nutrient-enriched canal water entering the Refuge in this area. Our analysis is capturing richness information at the macroscale level, thus details of each species presence

and abundance can not be estimated. However richness information is extremely useful to capture the behavior of the whole ecosystem.

References

[1] SFWMD (2000) Ecological effects of phosphorus enrichment in the everglades. chapter 3. In: Redfield G, editor, Everglades Consolidated Report., South Florida Water Management District.

[2] McVoy C, Said W, Obeysekera J, VanArman J, Dreschel T, editors (2011) Landscape and Hydrology of the Predrainage Everglades. University Press of Florida.

[3] Watts D, Cohen M, Heffernan J, Osborne T (2010) Hydrologic modification and the loss of self-organized patterning in the ridgeslough mosaic of the everglades. Ecosystems 13: 813-827.

[4] Bissonette J, Storch I, editors (2007) Temporal Dimensions of Landscape Ecology. University Press of Florida.

[5] Pope K (1989) Vegetation in relation to water quality and hydroperiod on the loxahatchee national wildlife refuge – msc thesis. Technical report, University of Florida, Gainesville.

[6] Childers D, Doren R, Jones R, Noe G, Rugge M, et al. (2003) Decadal change in vegetation and soil phosphorus pattern across the everglades landscape. J Environ Qual 32: 344362.

[7] Childers D, Boyer J, Davis S, Madden C, Rudnick D, et al. (2006) Relating precipitation and water management to nutrient concentrations in the oligotrophic "upside-down" estuaries of the florida everglades. Limnol Oceanogr 51: 602616.

[8] Brandt L, Kitchens W (1998) Spatial and temporal changes in tree islands of the arthur r. marshall loxahatchee national wildlife refuge in response to altered hydrologies. Technical report, Florida Cooperative Fish and Wildlife Research Unit, University of Florida.

[9] USFWS (1972) A report on fish and wildlife resources affected by water regulation schedule for loxahatchee national wildlife refuge, florida. Technical report, U.S. Department of Interior, U.S. Fish and Wildlife Service, Bureau of Sports Fisheries and Wildlife, Boynton Beach, FL.

[10] Warton DI, Wright IJ, Falster DS, Westoby M (2006) Bivariate line-fitting methods for allometry. Biological Reviews 81: 259–291.
